# Real‐Time Probing of Morphological Evolution and Recrystallization During Solvent Annealing in Blade‐Coated All‐Polymer Organic Solar Cells Using In Situ X‐Ray Scattering

**DOI:** 10.1002/advs.202501823

**Published:** 2025-06-23

**Authors:** Jialiang Hao, Yang Feng, Qianyi Ma, Hongxiang Li, Chunxia Hong, Chen Hou, Ying Wang, Yang Jing, Yiwen Li, Guangfeng Liu, Xiuhong Li, Aiguo Li, Fenggang Bian, Ruijie Ma, Yuanyang Wang, Yuying Huang, Chunming Yang

**Affiliations:** ^1^ Shanghai Synchrotron Radiation Facility Shanghai Advanced Research Institute Chinese Academy of Sciences Shanghai 201204 China; ^2^ School of Chemical Engineering and Technology Taiyuan University of Science and Technology Taiyuan 030027 China; ^3^ Institute of Flexible Electronics Northwestern Polytechnical University Xi'an 710100 China; ^4^ School of Materials Science and Engineering University of Science and Technology Beijing Beijing 100049 China; ^5^ College of Polymer Science and Engineering State Key Laboratory of Polymer Materials Engineering Sichuan University Chengdu 610065 China; ^6^ Shanghai Institute of Applied Physics Chinese Academy of Sciences Shanghai 201800 China; ^7^ University of Chinese Academy of Sciences Beijing 100049 China; ^8^ Department of Electrical and Electronic Engineering Research Institute for Smart Energy (RISE) Photonic Research Institute (PRI) The Hong Kang Polytechnic University Hong Kang 999077 China

**Keywords:** all‐polymer solar cells, blade‐coating, GIWAXS, in situ, solvent vapor annealing

## Abstract

Optimizing the morphology of the active layer is crucial for achieving high photovoltaic conversion efficiency in all‐polymer solar cells (APSCs). Solvent vapor annealing (SVA) is an essential post‐treatment strategy for controlling active layer morphology. However, most current SVA are conducted ex situ, limiting their ability to accurately reveal the morphological evolution of active layers of APSCs. In this study, in situ synchrotron radiation GIWAXS and in situ UV–vis spectroscopy combined with GISAXS is used to monitor the morphological evolution of PM6/PY‐IT blends during the SVA process in real‐time. Results showed that the PY‐IT absorption peak exhibited a red shift under a nonpolar carbon disulfide vapor, while a blue shift is observed during the SVA process with a polar chloroform vapor. The SVA process can be divided into three stages: solvent swelling, recrystallization, and molecular rearrangement. For thermally pre‐annealed samples subjected to chloroform SVA, the power conversion efficiency (PCE) increased by 15.1%. The improved PCE stems from reduced crystal plane spacing (d‐spacing), enhanced crystal coherence length, and optimal phase separation via SVA. Pre‐annealing suppresses excessive swelling, emphasizing the reordering dynamical role in the morphology of APSCs. This study offers insights into balancing SVA conditions to maximize performance and minimize adverse effects.

## Graphical abstract

In situ grazing‐incidence X‐ray scattering and in situ UV–vis spectroscopy are being used for the first time to reveal the morphological evolution of all‐polymer solar cell (APSCs) blends during solvent vapor annealing (SVA). SVA strongly influences the recrystallization and reordering behavior of APSCs blends, successfully establishing the relationship between morphological evolution and photovoltaic performance under different post‐treatment conditions.

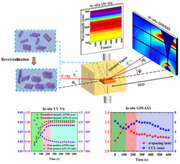

## 1 Introduction

All‐polymer solar cells (APSCs) fabricated with polymer donors and polymer acceptors have garnered significant attention due to their excellent thermal stability, material flexibility, and solution processability, making them promising candidates for lightweight, large‐area printed, and short‐energy payback applications in wearable and stretchable devices.^[^ ^1^, ^2^, ^3^, ^4^, ^5^ ^]^ To date, APSCs have achieved power conversion efficiencies (PCE) exceeding 20%.,^[^ ^6^, ^7^, ^8^ ^]^ Industrialization has a bright future. The efficiency of all‐polymer systems is also approaching the frontier of polymer small molecules with the help of continuous innovation in materials and continuous optimization of device processes.^[^ ^9^, ^10^, ^11^, ^12^, ^13^, ^14^, ^15^, ^16^ ^]^ In these efforts, rational morphology optimization to regulate the crystallization and phase separation of the donor is a crucial aspect, and therefore, morphology regulation will remain a key topic in the future development of all‐polymer systems.^[^ ^17^, ^18^ ^]^

Among the various methods to optimize the all‐polymer morphology, pre‐processing tools have received sufficient attention in the field,^[^ ^19^, ^20^ ^]^ including component engineering, solvent selection, additive development, etc. These tools have not only proved to be effective in improving the efficiency of the devices but also their corresponding principles of morphology optimization have been systematically investigated,^[^ ^21^, ^22^ ^]^ including powerful tools such as in situ characterization; however, equally important post‐processing tools are still lacking in systematic research, such as thermal annealing and solvent annealing, which are widely used.^[^ ^23^, ^24^ ^]^ Although it has been reported in the literature that Yao et al. implemented a two‐step annealing process (SVA+ Thermal annealing (TA)) to fine‐tune crystallinity and phase separation in spin‐coated PM6‐b‐PYIT block copolymer devices, increasing efficiency from 14.17% to 15.01%.^[^ ^25^ ^]^ Similarly, Zhang et al. applied a post‐processing approach (TA +SVA) combined with a solid additive strategy in the spin‐coated PM6:PY‐IT blend films, which effectively traps incident light in the active layer, accelerates exciton‐to‐polaron conversion, and significantly improves light‐to‐electricity conversion efficiency from 15.17% to 19.06%.^[^ ^6^ ^]^ but there is no systematic and comprehensive in situ characterization to reveal the whole process of morphology optimization and provide sufficient theoretical guidance for future studies.

**Figure 1:**
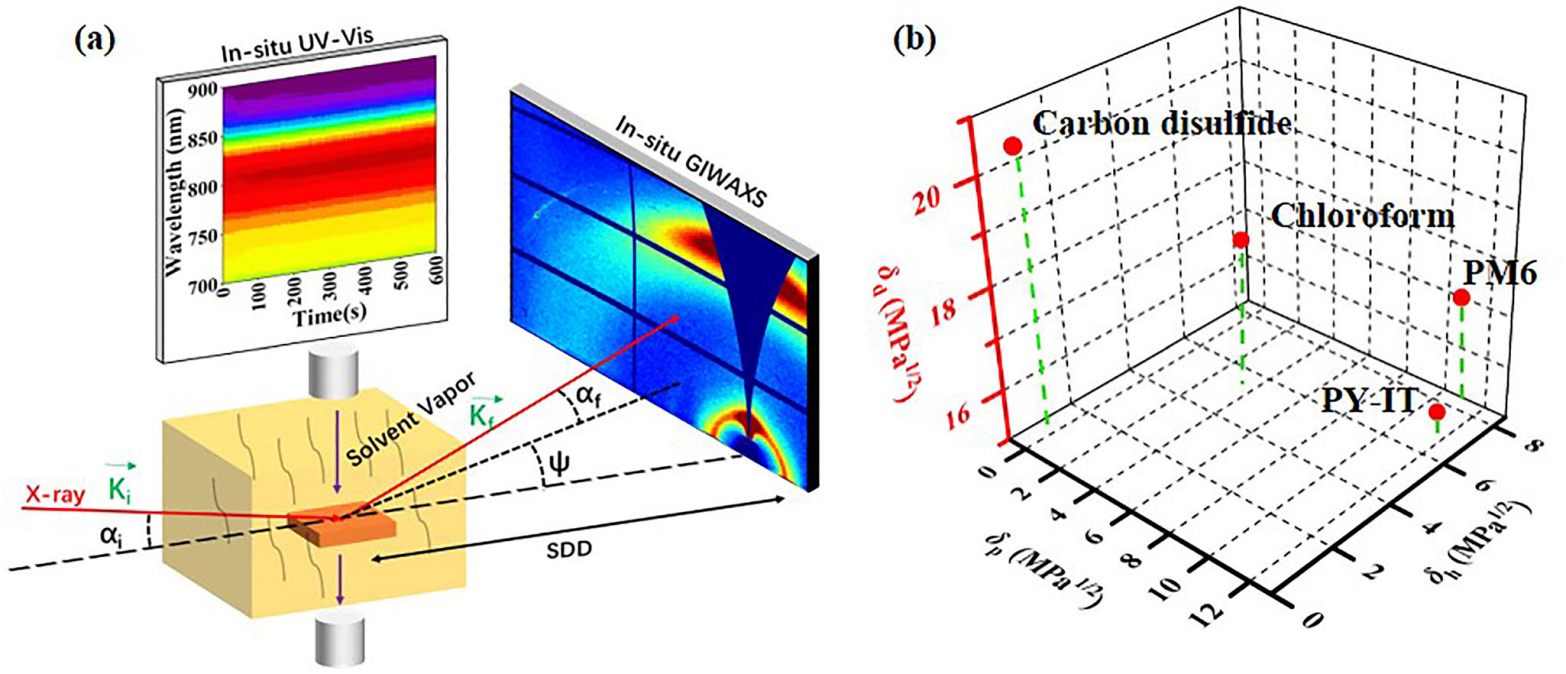
a) Schematic diagram of in situ GIWAXS and in situ UV–vis measurement setup. b) Solubility parameter coordinates of PM6, PY‐IT, CF, and CS_2_.

Grazing‐incidence wide‐angle X‐ray scattering (GIWAXS) is a powerful technique that enables in situ, real‐time probs the morphology evolution of thin films on an atomic scale.^[^ ^26^ ^]^ In this work, we applied in situ GIWAXS and in situ ultraviolet‐visible (UV–vis) absorption spectroscopy to investigate the morphology evolution of blade‐coated PM6/PY‐IT blend films during SVA in **Figure** **1**a.^[^ ^27^, ^28^, ^29^, ^30^, ^31^, ^32^ ^]^ The study revealed three stages in the SVA process: solvent swelling, recrystallization, and molecular rearrangement. Recrystallization reduced the d‐spacing, increased the crystal coherence length (CCL), and established an optimal phase separation size, thereby enhancing device performance. Through a comparative study of the polar solvent Chloroform (CF) and the non‐polar solvent Carbon disulfide (CS₂), it was found that the polymer alkyl side chains are subjected to a stronger force by the polar solvent CF. Consequently, the amorphous chains are depleted, leading to a closer donor‐acceptor interaction and an enhancement in charge transfer efficiency. However, excessive SVA could lead to crystal re‐swelling during molecular rearrangement, which hampers performance, while preheating mitigates this effect. To our knowledge, this work is the first to elucidate the dynamic mechanisms of SVA in controlling APSC morphology through in situ monitoring.^[^ ^33^, ^34^ ^]^

## 2 Results and Discussion

### 2.1 Solvent Vapor Selection Principles

In order to predict the polymer‐solvent interaction, the solubility parameter *δ_t_* was first proposed by J.H. Hildebrand in 1916. Later, C. M. Hansen proposed that *δ_t_* consists of dispersion forces (*δ_d_*), dipole chain forces (*δ_p_*), and hydrogen bonding forces (*δ_h_*) as shown in the conversion equation:^[^ ^35^ ^]^

(1) $$\delta_{t}^{2}=\delta_{d}^{2}+\delta_{p}^{2}+\delta_{h}^{2}$$

The dipole chain forces and the partial derivatives of hydrogen bonding can be determined theoretically and measured experimentally. After obtaining the parameters for both combinations, the remainder of the Hildebrand parameter becomes the parameter for the dispersion force.^[^ ^35^ ^]^ If these three parameters are obtained, interchain compatibility can be measured quantitatively. The Hansen solubility parameter, distance (*R_a_*), is the most valuable parameter for quantifying the compatibility of two molecules, for example, the solubility of a polymer in a solvent; denoted as:

(2) $$R_{a}^{2}=4{\left(\delta_{d1}-\delta_{d2}\right)}^{2}+{\left(\delta_{p1}-\delta_{p2}\right)}^{2}+{\left(\delta_{h1}-\delta_{h2}\right)}^{2}$$

A lower *R_a_* indicates better intermolecular compatibility. This theory allows the solubility of the solvent and the interaction between the polymer and the solvent to be calculated quantitatively.

Since the solubility and saturation vapor pressure of different solvents vary, and the main and side chains in all‐polymer blended films are susceptible to the vapor forces of different solvents, it is necessary to evaluate the solubility and saturation vapor pressure of the selected annealing solvents.^[^ ^36^, ^37^, ^38^ ^]^ The saturated vapor pressures of the solvents were determined using the solvent Antoine's coefficient table, with the results presented in Tables S1 and S2 (Supporting Information). The solubility of the donor/acceptor in various solvents was measured through the solubility experiments. These experiments revealed that CF exhibited the highest solubility, reaching 27.0 mg mL^−1^ for the polymer donor PM6 and 22.6 mg mL^−1^ for CS_2_. For the polymer acceptor PY‐IT, the solubility was 102.6 mg mL^−1^ in CF and 79.5 mg mL^−1^ in CS_2_.To further validate the accuracy of the solubility experiments, and assess the microscopic forces of solvents on the donor/acceptor, the interaction parameters *R_a_* was used to quantitatively analyze the interactions between PM6, PY‐IT, and the solvents CF and CS_2_ solvents. The solubility parameters of PM6 and PY‐IT were calculated using the HOY method. Equations S1–S10 provide the equations of the system, including the molar functions, auxiliary formulas, *δ_t(total)_* and final expressions for each component of *δ*. The incremental values of the molar attraction function are listed in Table S3 (Supporting Information). Ft represents the molar attraction function, *F_p_* is the polar component, *V* is the molar volume of the polymer structural unit, and *Δ_T_^(P)^* is the correction used in the auxiliary equation.^[^ ^35^ ^]^ Ultimately, the values of *δ_d_*, *δ_p_*, and *δ_h_* were calculated for the solvent and PM6, PY‐IT. The detailed properties and parameters for the solvents, PM6, and PY‐IT are listed in Table S4 (Supporting Information). Ra was calculated according to Equation (2) as shown in Table S4 (Supporting Information). The solubility parameters coordinate for PM6, PY‐IT with each solvent were given in Figure 1b. In the plot, the closer the solvent is to PM6 or PY‐IT, the smaller *R_a_* value, indicating stronger molecular interaction. The results were noteworthy: *R_a_* for CF was smaller for both PM6 and PY‐IT, aligning well with the results of the solubility experiment. The differences in *δ_d_*, *δ_p_* and *δ_h_* for CF were smaller than those for CS_2_, suggesting that CF exhibits stronger interaction with the donor‐acceptor pair compared to CS_2_.

### 2.2 Device Performance

**Figure 2:**
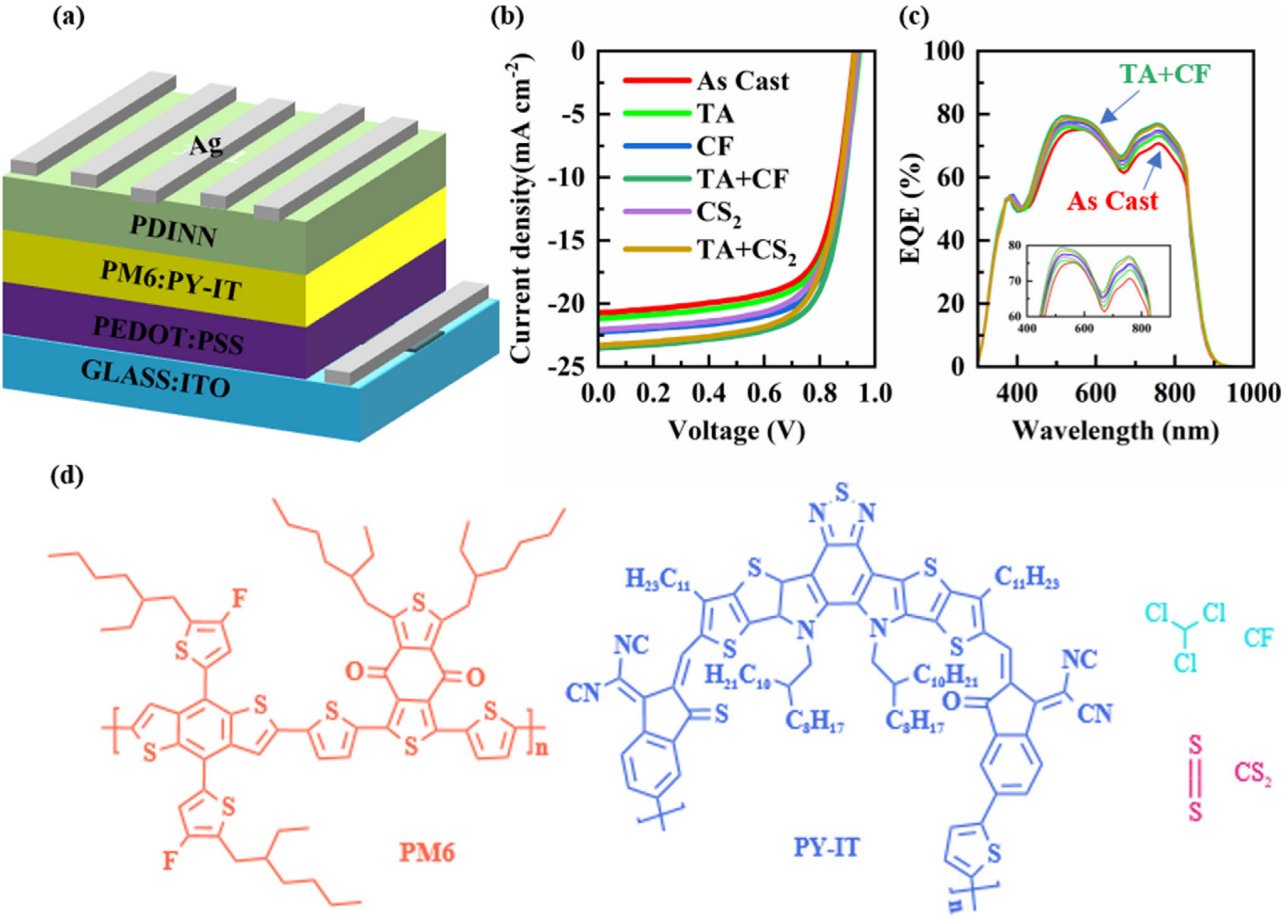
a) Schematic structure of the devices; b) The *J–V* curves for APSC devices under various post‐treatment conditions;c) The EQE curves for APSC devices under various post‐treatment conditions. d) The chemical structures of PM6, PY‐IT, CF, and CS_2_.

To study the effects of different post‐treatment strategies on device performance, four methods were implemented: CF and CS_2_ SVA, with and without TA pretreatment. The samples are labeled as CF, TA+CF, CS_2_, and TA+CS_2_. **Figure** **2**a shows the device structure optimized for this study, and Figure 2b along with **Table** **1** summarizes the current density‐voltage (*J–V*) curves and parameters achieved under each treatment. Among them, the CS_2_‐treated device showed a lower PCE of 14.35%, with open‐circuit voltage (*V_oc_*) of 0.93 V, current density (*J_sc_*) of 22.47 mA cm^−^^2^, and fill factor (*FF*) of 67.80%. However, with TA pretreatment, the PCE improved slightly to 14.78%. Interestingly, the TA+CF treated device achieved the highest PCE of 15.42% with an enhanced *J_sc_* of 23.53 mA cm^−^^2^, which suggested that moderate phase separation and crystallinity in the presence of high solubility CF solvents are very beneficial in achieving efficient charge transport.^[^ ^20^ ^]^This PCE is comparable to the best previously reported performance for spin‐coated binary devices.^[^ ^39^ ^]^

**Table 1:** APSC uses different solvents and pretreated SVA for PV and EQE parameters.

Treatment	*V_oc_* [v]	*J_sc_* [mA cm^−2^]	*FF*[%]	PCE[Average ^c)^]
As Cast	0.92 (0.92 ± 0.01)	21.12 ^a^ ^)^ /20.11 ^b^ ^)^ (21.12±0.79)^a^ ^)^	68.77 (68.77 ± 1.12)	13.40 (13.40 ± 0.17)
TA	0.93 (0.93 ± 0.01)	21.19 ^a^ ^)^ /20.25 ^b^ ^)^ (21.19 ± 0.64)^a^ ^)^	68.03 (68.03 ± 0.83)	13.54 (13.54 ± 0.26)
CF	0.93 (0.93 ± 0.01)	22.24 ^a^ ^)^ /21.46 ^b^ ^)^ (22.24 ± 0.49)^a^ ^)^	70.72 (70.72 ± 0.71)	14.52 (14.52 ± 0.23)
TA+CF	0.93 (0.93 ± 0.01)	23.53 ^a^ ^)^ /22.84 ^b^ ^)^ (23.53 ± 0.44)^a^ ^)^	70.03 (70.03 ± 1.24)	15.42 (15.42 ± 0.37)
CS_2_	0.93 (0.93 ± 0.01)	22.47 ^a^ ^)^ /21.75 ^b^ ^)^ (22.47 ± 0.31)^a^ ^)^	67.80 (67.80 ± 1.16)	14.35 (14.35 ± 0.16)
TA+CS_2_	0.93 (0.93 ± 0.01)	23.29 ^a^ ^)^ /22.57 ^b^ ^)^ (23.29 ± 0.29)^a^ ^)^	68.52 (68.52 ± 1.03)	14.78 (14.78 ± 0.24)

^a)^ *J_SC_* obtained from *J‐V* curves measurements;

^b)^ *J_SC_* obtained from the EQE tests;

^c)^ Average PCE and deviation values were calculated over 15 devices.

The external quantum spectrum (EQE) spectra of the best device of each type were measured to screen the reliability of the device test results. Figure 2b shows the current density curves achieved under each treatment. The integrated *J_SC_* values were also listed in Table 1, showing deviations within 5% compared to those derived from the *J–V* curves. Notably, the EQE curves of PM6:PY‐IT treated with TA+CF exhibit the highest EQE response. However, it is worth noting that the shoulder peaks in the donor/acceptor region for TA+CF are weaker than those observed with other post‐processing treatments. This suggests that TA+CF strategy may more effectively reduce the aggregation of PM6 and PY‐IT, thereby enhancing the photoelectric conversion efficiency.^[^ ^40^ ^]^

### 2.3 Morphological Characterization of AFM and TEM

**Figure 3:**
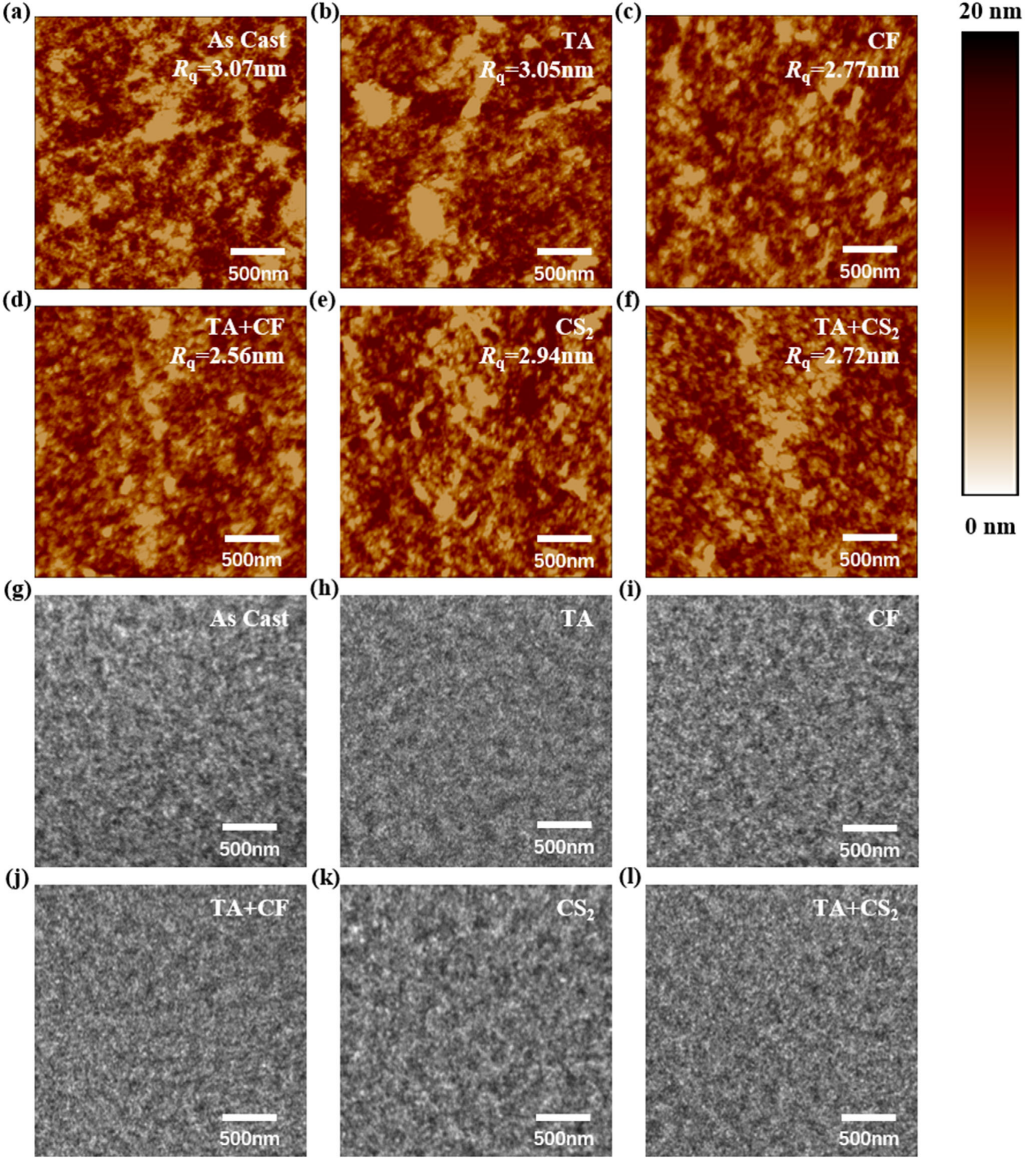
AFM a‐f) and TEM images g‐l) of films treated under different post‐treatment conditions.

**Figure** **3**a–f showed the Atomic Force Microscope (AFM) images, revealing that films treated with CS_2_ exhibit an uneven morphology with larger grain sizes, resulting in a relatively high root mean square roughness (*R_q_*) of 2.94 nm in Figure 3e. This uneven grain size distribution may increase interfacial resistance, hindering charge transport and thus affecting device performance.^[^ ^41^ ^]^ The *R*_q_ value of the film treated with TA+CS_2_ is 2.72 nm in Figure 3f, and films subjected to TA treatment generally exhibit lower *R_q_* values than those without treatment, suggesting that TA treatment positively influences film morphology to the film treated with CF alone, which has an *R_q_* of 2.77 nm in Figure 3c, the TA+CF‐treated blend film forms a smaller and more continuous surface morphology, achieving a lower *R_q_* value of 2.56 nm in Figure 3d. Studies have shown that lower *R_q_* values help improve interfacial stability and reduce interfacial resistance.^[^ ^42^ ^]^ As Transmission Electron Microscope(TEM) images shown in Figure 3g–l, films treated with CS_2_ exhibit a large and discontinuous phase‐separated morphology and domain structure, which is unfavorable for exciton dissociation at the donor/acceptor interface in Figure 3k.^[^ ^43^ ^]^ Films subjected to TA treatment also show smaller domain sizes and phase‐separated morphology compared to untreated films, corroborating the AFM observations. Similarly, under the combined TA+CF treatment, the active layer film exhibits a more uniform morphology in Figure 3j, forming a well‐interconnected network structure conducive to exciton dissociation and charge transport, reducing charge recombination and improving charge transport efficiency.^[^ ^44^ ^]^

### 2.4 GIWAXS Structural Characterization

GIWAXS and GISAXS (Grazing Incidence Small Angle X‐ray Scattering) can be used together to comprehensively characterize the microstructure of the active layer of all‐polymer solar cells at different scales (from atomic level to nanometer). GIWAXS: mainly probes the crystalline structure at atomic scale, such as crystal plane spacing, π‐π stacking orientation, CCL, and relative crystallinity, etc., and is suitable for analyzing the arrangement of molecular chains and crystal growth direction (e.g., out‐of‐plane (OOP) or in‐plane orientation (IP));^[^ ^45^ ^]^GISAXS: focuses on nanoscale phase separation structure, such as the size of the donor‐acceptor phase region, the uniformity of the phase distribution, and the formation of interpenetrating networks, which can reveal the phase separation characteristics of the donor and acceptor and the interfacial morphology in the active layer. Therefore, the synergistic use of GIWAXS and GISAXS can cover the multi‐scale information from molecular arrangement (≈0.1 nm) to nano‐phase region (≈100 nm), and comprehensively analyze the mechanism of the influence of the active layer morphology on the device performance.^[^ ^46^, ^47^ ^]^

**Figure 4:**
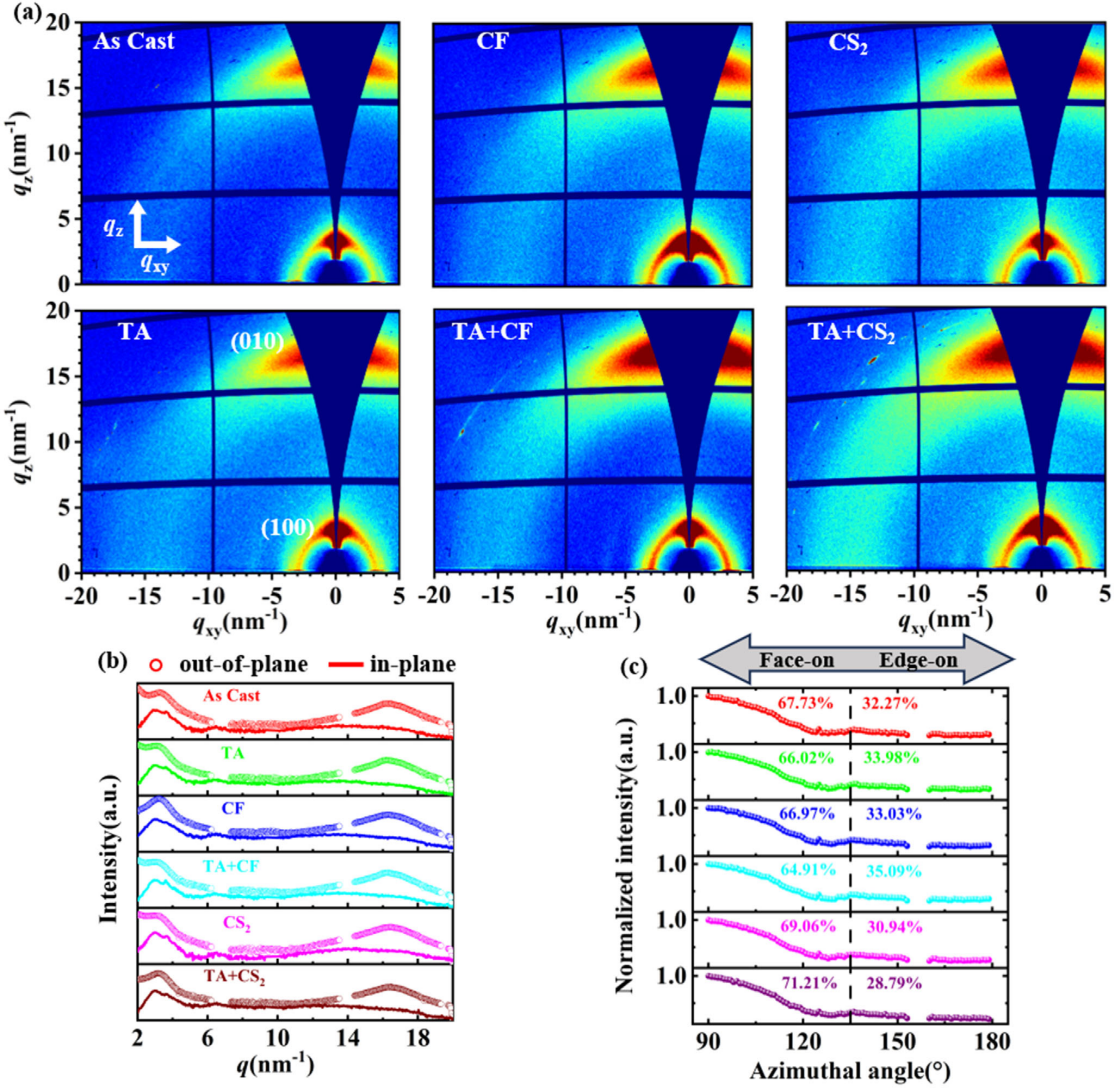
a) 2D GIWAXS patterns of PM6: PY‐IT films with different SVA solvents and pretreatments; b) OOP and IP scattering profiles derived from the GIWAXS patterns; c) Intensity‐corrected pole figures of the (010) peak.

To analyze the effects of post‐treatment on the crystallinity of the active layer, GIWAXS was used for further investigation.^[^ ^48^, ^49^ ^]^ The 2D GIWAXS patterns are shown in **Figure** **4**a. It is observed that the (100) peak corresponding to PM6, at q≈2.94 nm^−1^, becomes sharper with TA+CF treatment, indicating a more compact and ordered molecular arrangement in PM6. The morphological parameters obtained through Gaussian fitting are summarized in Figure S4 and Table S6 (Supporting Information). The CCL was calculated using the Scherrer equation.^[^ ^50^ ^]^ For blend films treated with CF, TA+CF, CS_2_, and TA+CS_2_, the CCL for the (100) lamellar peak in the OOP direction were 5.7, 3.4, 5.6, and 3.8 nm, respectively. The significant reduction in CCL with TA pretreatment suggests that excessive crystallinity could hinder effective exciton dissociation, explaining the lower PCE observed in devices without TA pretreatment.^[^ ^51^ ^]^ As shown in Table S4 (Supporting Information), the smaller dispersive force (*δ_d_*) difference of the CF solvent on the donor/acceptor results in a greater impact on the alkyl side chain. Consequently, the CF‐treated films exhibit a smaller CCL compared to those treated with CS_2_. Figure 4c showed orientation information derived from azimuthal integration profiles.^[^ ^52^, ^53^ ^]^ Peaks near χ = 90° (vertical cut) and χ = 180° (horizontal cut) correspond to face‐on and edge‐on orientations, respectively. The fractions of face‐on orientation for films treated with CF, TA+CF, CS_2_, and TA+CS_2_ were 67.0%, 64.9%, 69.1%, and 71.2%, respectively. For CS_2_‐treated films, the face‐on orientation fraction showed an increasing trend with TA pretreatment, contrasting with CF‐treated films. The difference between the pretreatments of CF and CS_2_ should be attributed to the polar solvent CF, which has smaller differences in dipole chain force (*δ_p_*) and hydrogen bonding (*δ_h_*) compared to CS_2_. This results in stronger interactions with the main chains of the film. In contrast, the non‐polar solvent CS_2_ has the opposite effect, disrupting the orientation of film and reduces face‐on π‐π stacking. SVA can enhance crystalline order while maintaining the initial favorable orientation, avoiding large‐scale reorientation, thus preserving the initial favorable orientation. Previous studies have demonstrated that a mixture of face‐on and edge‐on orientations facilitates the formation of 3D charge pathways, promoting efficient charge transport.^[^ ^54^, ^55^ ^]^ Additionally, CF solvents with high solubility provide a greater driving force for recrystallization and phase separation.^[^ ^56^ ^]^

### 2.5 GISAXS Structural Characterization

**Figure 5:**
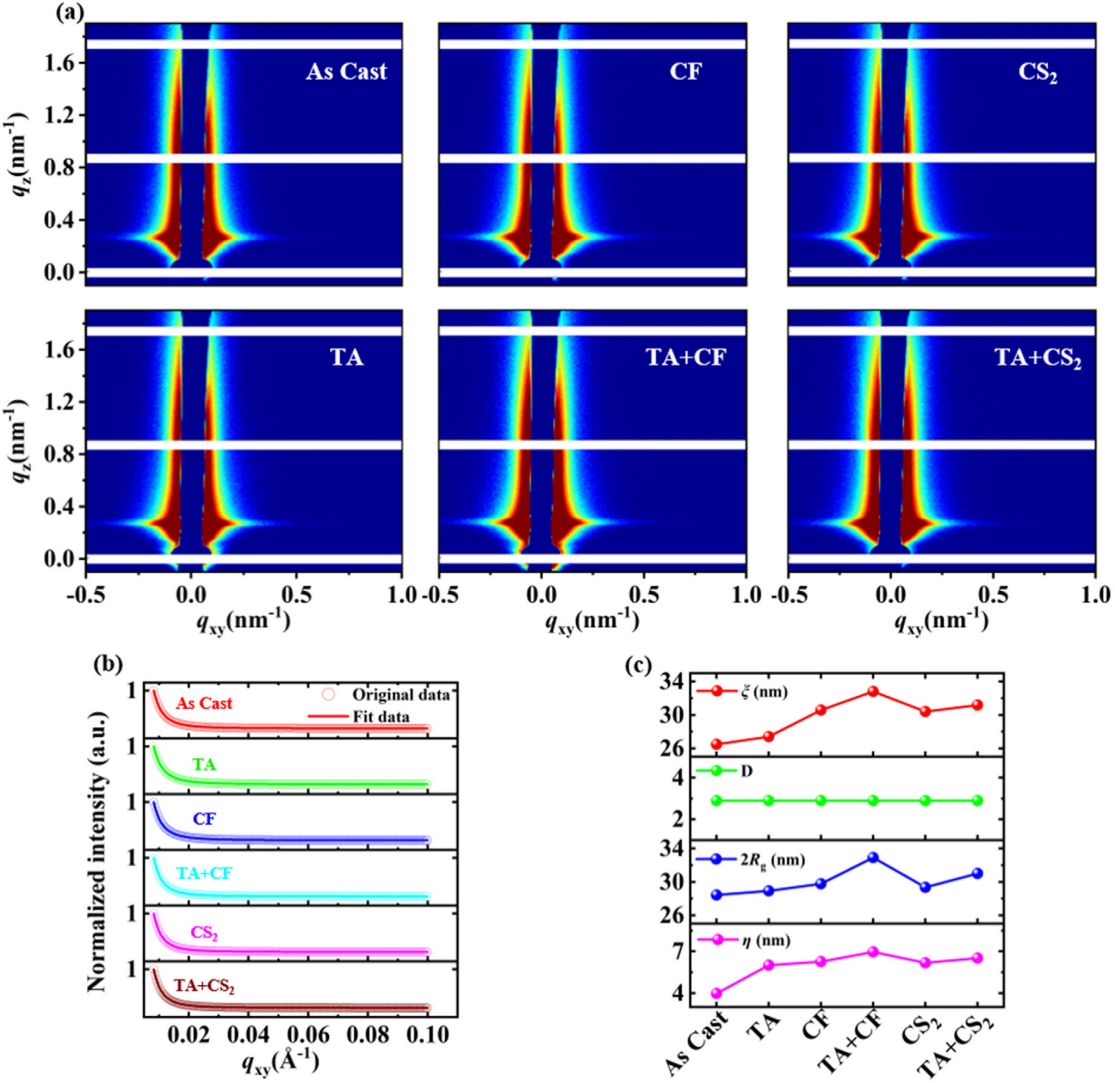
a) 2D GISAXS patterns of PM6:PY‐IT films with various SVA solvents and pretreatment; b)1D profiles derived from GISAXS patterns. c) The correlation length of the donor (*ξ*), fractal dimension (D), the correlation length (*η*) and domain size of acceptor aggregation (2*R_g_*) of PM6:PY‐IT blend films with various SVA solvents and pretreatment.

The GISAXS measurements were conducted on a mesoscale level to further investigate nanoscale phase separation behavior.^[^ ^57^, ^58^, ^59^ ^]^ The obtained 2D patterns in **Figure** **5**a and 1D profiles (open circles in Figure 5b) from the integration of Figure 5a provide insights into the morphology. Using a universal model based on the Distorted Wave Born Approximation (DWBA) for effective interface fitting (Supporting information), key parameters were extracted, such as the donor correlation length (*ξ*), the size of acceptor aggregates (2*R_g_*), the fractal dimension of the acceptor (D), and the acceptor correlation length (*η*).^[^ ^60^ ^]^ These parameters are summarized in Figure 5c and Table S9 (Supporting Information). Notably, the blend films treated with CF, TA+CF, CS_2_, and TA+CS_2_ exhibited ξ values of 30.6, 32.8, 30.4, and 31.2 nm, respectively. The TA+CF‐treated blend film showed the largest values for *ξ*, 2*R_g_*, and *η*. Conversely, CS_2_ treatment alone led to a smaller increase in *ξ*, but a noticeable increase in the acceptor correlation length following TA pretreatment, suggesting that both TA and SVA treatments enhance acceptor aggregation. This increase in the domain size of PY‐IT acceptor aggregates, driven by pre‐TA treatment, helps achieve appropriate crystallinity and phase separation, thus facilitating efficient charge separation and transport.^[^ ^61^, ^62^ ^]^

### 2.6 In Situ UV–Vis Characterization

**Figure 6:**
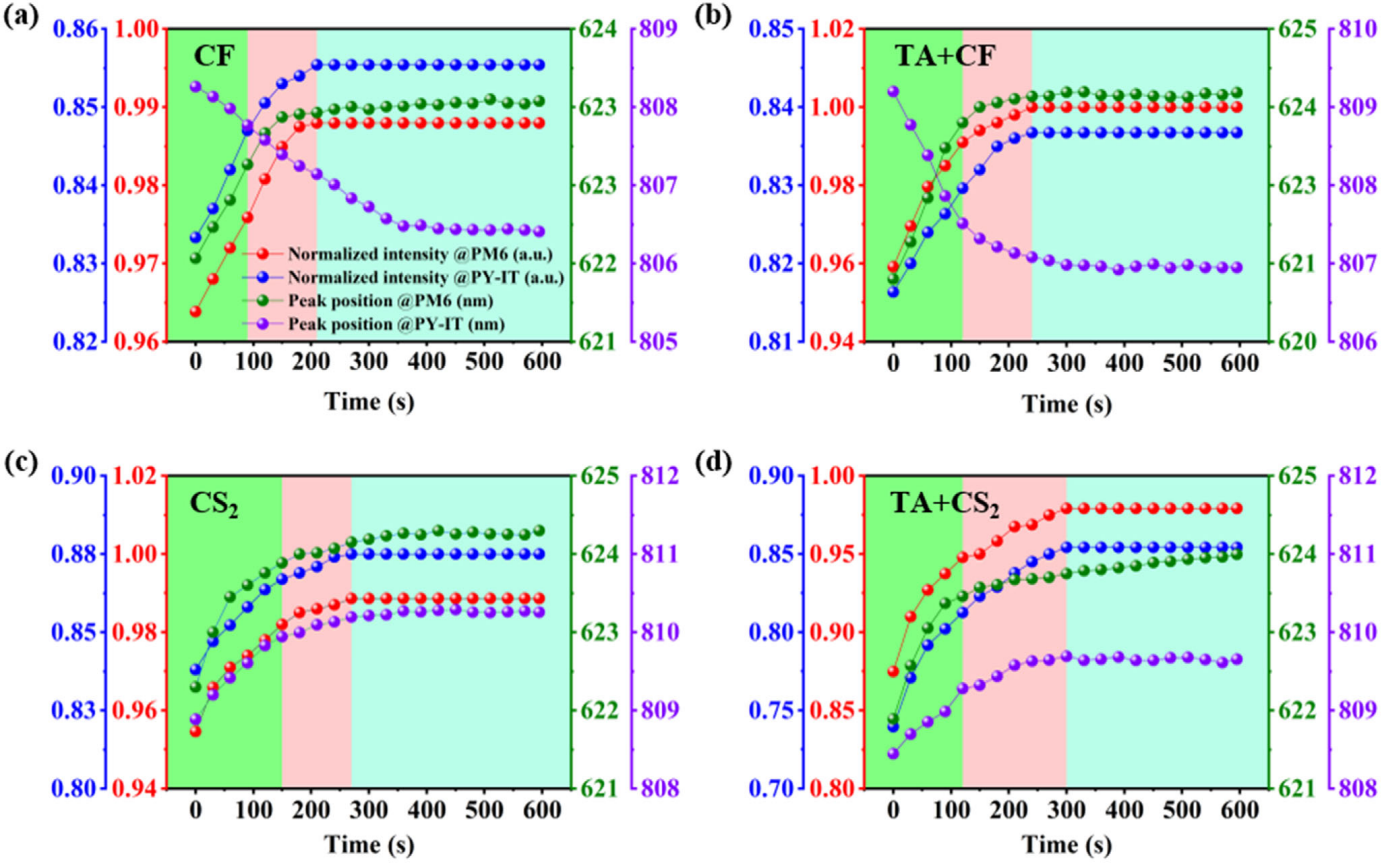
Time‐dependent evolution of the PM6 and PY‐IT peak intensities and positions in blend films under different treatment: a) SVA:CF, b)TA+SVA:CF, c) SVA:CS_2_, and d) TA+SVA:CS_2_. The data were obtained from in situ UV–vis measurements.

To comprehensively analyze the impact of SVA on the active layer morphology, in situ UV–vis spectroscopy was employed to observe molecular conformation transitions and rearrangements, reflecting crystallization dynamics within the active layer.^[^ ^63^, ^64^ ^]^ As shown in Figure S2 (Supporting Information), the characteristic absorption peaks of PM6 and PY‐IT appear at ≈600 and 835 nm, respectively. Based on the peak position and intensity changes, the SVA process can be divided into three stages: the preparatory phase of recrystallization under solvent vapor (highlighted in green in **Figure** **6**), the crystalline restructuring under solvent vapor (highlighted in pink) and the equilibrium state after restructuring (highlighted in cyan). As shown in Figure 6, there was a noticeable change in the slope of the UV peak shift around the 100‐s mark, which correlates with the d‐spacing variations observed in the GIWAXS results. This suggests that while a distinct UV peak shift may not be immediately apparent, the change in slope indicates an underlying structural transformation consistent with the GIWAXS data, this serves as a basis for differentiation between the first and second phases. It also serves as a basis for differentiation between phases II and III when the donor/acceptor absorption intensity reaches stabilization. The different saturation vapor pressures of the solvents and solubility of materials may significantly influence the SVA process.^[^ ^56^, ^65^, ^66^ ^]^Therefore, to understand the effect of solvent on the active layer film during SVA, the saturation vapor pressures and solubility parameters of two highly volatile solvents (CF and CS_2_) were estimated, as listed in Tables S1 and S2 (Supporting Information). It was observed that CF exhibits higher solubility for PM6 and PY‐IT compared to CS_2_, while CS_2_ has a higher saturation vapor pressure for these materials than CF (Table S2, Supporting Information). By analyzing the in situ absorption versus solubility plot in Figure S3 (Supporting Information), the morphology of the blended films transitions rapidly to the absorption‐enhanced stage was observed under the lower saturation vapor pressure and higher solubility conditions of CF. Whereas, a slower evolution of absorption enhancement was observed under CS_2_ treatment, characterized by higher saturation vapor pressure and lower solubility. These findings suggested that high solubility facilitates the absorption transition of the films at lower saturated vapor pressure. Interestingly, we found for the first time that under solvent vapor treatment with either CF or CS_2_, the absorption intensity of the pre‐TA‐treated blend film reached a stable state relatively slowly, as shown in Figure 6b,d This delay indicates that pre‐TA treatment induced a degree of crystal reorganization, requiring more time to reach equilibrium during the SVA process. This adjustment helped the system achieve an optimal phase separation state more efficiently during SVA. The synergistic effect of TA pretreatment and SVA enhances a more stable face‐on orientation and appropriate phase separation sizes, as demonstrated by the GIWAXS and GISAXS results.^[^ ^67^ ^]^

Compared to the SVA process using CS_2_, the absorption peaks of PM6 and PY‐IT exhibited a more pronounced shift during the SVA process with CF. Interestingly, under CS_2_ solvent vapor, the PY‐IT absorption peak gradually shifted to a longer wavelength, indicating a red shift. Whereas, during SVA process with CF, the PY‐IT absorption peak shifted to shorter wavelengths, demonstrating a blue shift. The blue shift suggested that under polar solvent vapor of CF, the donor molecules adopt a more compact conformation, likely due to the insertion of PY‐IT molecules into the PM6 matrix. This morphological change results in a lower‐energy charge transfer state, which is favorable for enhanced hole transport.^[^ ^68^ ^]^ In contrast, within the nonpolar CS_2_ vapor, the observed redshift was unfavorable for the forming a compact charge‐transfer state.

### 2.7 In Situ GIWAXS Characterization

**Figure 7:**
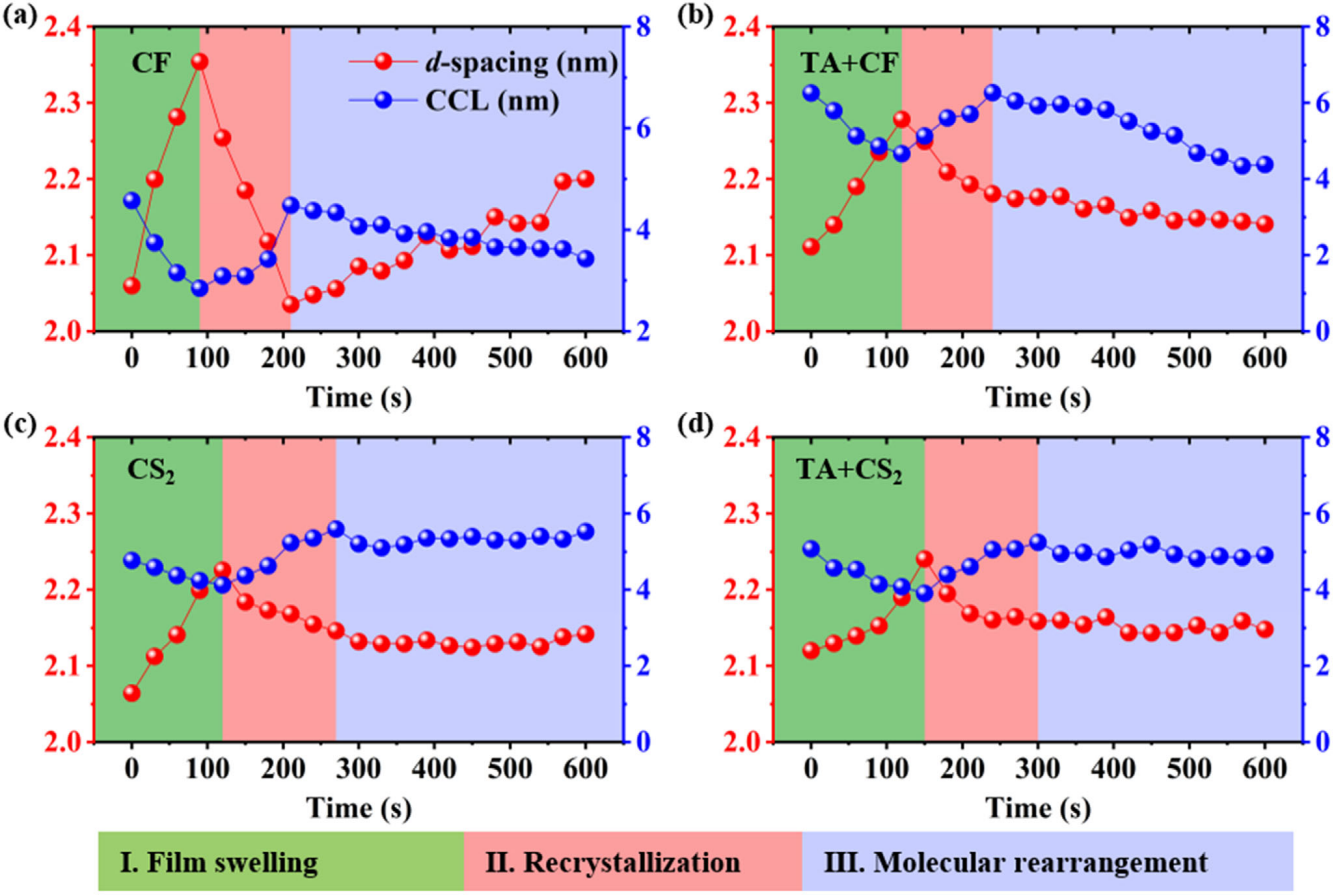
Evolution of d‐spacing and CCL evolution of (OOP)100 peak for blade coated PM6: PY‐IT blend films with a) CF, b)TA+ CF, c) CS_2_, and d) TA+ CS_2_. Data were obtained from in situ GIWAXS measurements.

The crystalline evolution of blended films under different post‐treatment conditions (with or without TA) was characterized using in situ GIWAXS during the SVA process.^[^ ^69^, ^70^, ^71^, ^72^ ^]^ The time‐dependent contour plots of the (100) and (010) peaks in the OOP and IP directions are shown in **Figure** **7**, Figures S6, and S7 (Supporting Information). By fitting the scattering peaks in the OOP and IP directions with Gaussian functions, parameters such as d‐spacing and CCL were analyzed, as illustrated in Figure 7, Figures S6, and S7 (Supporting Information). The SVA process can be divided into three stages based on the evolution of d‐spacing and CCL of the (OOP)100 peak, as shown in Figure 7: I) film swelling state, II) recrystallization state, and III) molecular rearrangement state.

As shown in Figure 7, during the first stage, the low boiling point of the annealing solvent causes a large number of solvent molecules to rapidly diffuse to saturation in both the atmosphere and the film, penetrating its interior and leading to film swelling. This behavior should be attributed to solvent‐side chain interactions that solvate the polymer side chains, resulting in an increase in d‐spacing and decreased CCL. These changes create additional free space, facilitating the reorientation and mobility of amorphous chains.^[^ ^73^ ^]^ Furthermore, as shown in Figure S10 (Supporting Information), no significant changes were observed in the π‐π stacking peaks, indicating that the interaction between the solvent and side chain is stronger than that between the solvent and the main chains. In the second stage, the d‐spacing decreases while CCL increases to formed a stable crystal by the side chains, as solvent molecules provide the necessary driving force for molecular reordering and recrystallization.^[^ ^74^ ^]^

In the third stage, the evolution whether TA pretreatment was applied and on the type of solvent vapor used. The CCL decreased with time under a polar solvent vapor of CF, while the value of CCL remained almost constant under a nonpolar solvent vapor of CS_2_. Compared with the solubility parameter *R_a_* of CS_2_, the CF solvent has a smaller solubility parameter *R_a_* for the donor and acceptor, which in turn has a larger solubility and can provide a larger mobility for molecular movement. Furthermore, the CF solvent has a smaller dispersion force difference of *Δδ_d_*, indicating that the CF solvent has a stronger force for the alkyl side chains. It should lead to changes of larger d‐spacing and CCL in the CF‐treated films than that with CS_2_ during this stage. In the case with the polar solvent vapor of CF, the d‐spacing increased in untreated films Figure 7a suggesting that excessive SVA can cause crystal reswelling again, while that of TA‐pretreated films exhibited a decrease in Figure 7b. Re‐swelling due to prolonged solvent exposure results in an excessive increase in d‐spacing and a decrease in CCL, leading to a disordered molecular arrangement that hinders charge transport. This phenomenon explains the reduction in PCE after reaching its peak as SVA duration increases in Tables S10–S13 (Supporting Information). The time‐dependent PCE data in Tables S10–S13 (Supporting Information) also show that the highest PCE aligns closely with the end of the second stage.

In the case of the polar solvent vapor of CF, the d‐spacing increased in untreated films in Figure 7a, suggesting that excessive SVA can lead to crystal reswelling. In contrast, the d‐spacing of TA‐pretreated films decreased in Figure 7b. Prolonged solvent exposure during SVA results in excessive d‐spacing expansion and a reduction in CCL, causing a disordered molecular arrangement that impairs charge transport. This effect accounts for the decline in PCE observed after it reaches its peak with increasing SVA duration in Tables S10–S13 (Supporting Information). Furthermore, the time‐dependent PCE data in Table S10–S13 indicate that the highest PCE closely corresponds to the end of the second stage.

Comparing the results of films without TA pretreatment in Figure 7a,c and with TA pretreatment in Figure 7b,d, we observe that both d‐spacing and CCL values are higher at the initial state (marked as “0” on the *x*‐axis) in TA‐pretreated films. This suggests that TA pretreatment induces recrystallization in the active layer. Additionally, TA pretreatment reduces the extent of d‐spacing increase in the first stage and the extent of decrease in the second stage, indicating that TA‐induced recrystallization improves the stability of the active layer morphology. Interestingly, due to this enhanced stability, the d‐spacing in TA‐pretreated films gradually decreases over time in the third stage, preventing the re‐swelling observed in Figure 7a. Meanwhile, in situ GISAXS results show that SVA appropriately enhances donor‐acceptor phase separation, highlighting the necessity of TA pretreatment for improving interfacial stability.

**Figure 8:**
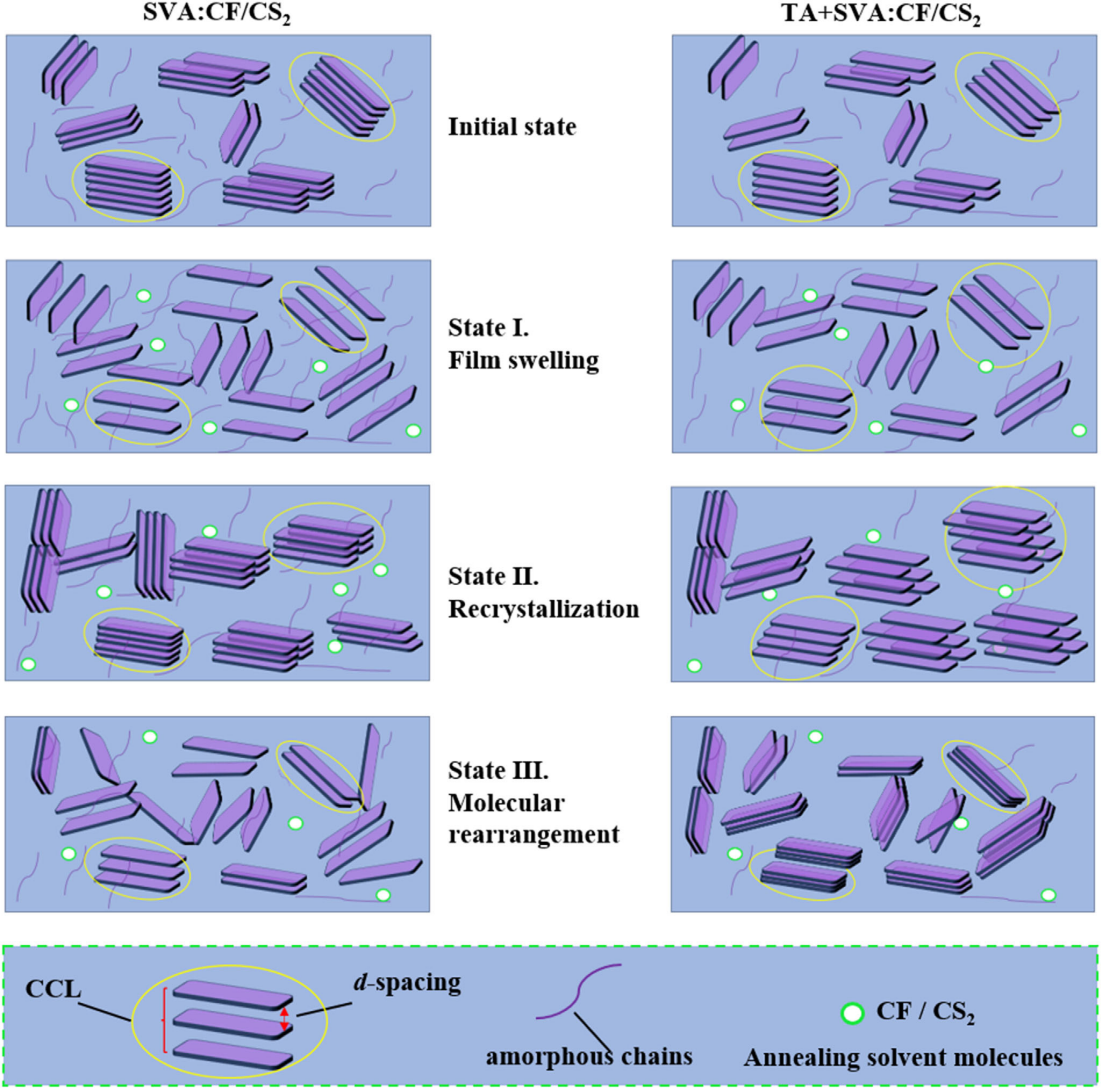
Mechanism diagrams illustrating the morphological evolution of all polymer OSCs during SVA (left) and the combined process of TA+SVA (right). (The yellow ellipses: CCL, green circles: solvent vapor, purple squares: crystalline chains, and purple lines: amorphous chains).

**Figure** **8** illustrates the mechanism of microstructure evolution during SVA and the combined TA+SVA treatment. The yellow ellipses represent CCL, the green circles indicate solvent vapor, the purple squares denote crystalline chains, and the purple lines correspond to amorphous chains. In the initial phase, the TA‐pretreated active layer film exhibits larger d‐spacing and CCL. During the first stage, the film swells, causing d‐spacing to continue increasing while CCL decreases. Once the d‐spacing expands to a certain threshold, amorphous chains transition from a frozen state below the glass transition temperature to a free state. Compared to films treated only with SVA, the TA+SVA‐treated films show smaller changes in both d‐spacing and CCL, indicating that the pretreated films are more stable. In the recrystallization phase (second stage), the d‐spacing starts to decrease, CCL increases, and crystallinity is further enhanced in Figure 4a. Compared to samples without TA pretreatment, the TA+SVA samples exhibit smaller variations in d‐spacing and CCL, preserving sufficient pathways for charge transport and improving the efficiency of photoelectric conversion. Therefore, the end of the second stage often corresponds to the peak PCE in Tables S10–S13 (Supporting Information). With continued SVA treatment, a third stage of molecular rearrangement occurs. Interestingly, in TA+SVA‐treated films, d‐spacing gradually decreases, displaying a trend opposite to that of non‐TA‐treated samples and showing greater stability against over swelling during solvent annealing. To sum up, the TA+SVA approach effectively stabilizes the active layer morphology by promoting orderly molecular rearrangement and preventing excessive swelling. This results in an optimal morphology with smaller d‐spacing, enhanced crystallinity, and appropriate phase separation sizes, thereby improving charge separation and transport.

## 3 Conclusion

In summary, we conducted a systematic investigation of the morphological evolution of blade‐coated all‐polymer blend films during the SVA process using in situ UV–vis absorption spectroscopy and in situ GIWAXS. In situ UV–vis results reveal that the intensities and peak positions of the donor and acceptor molecules in the polymer blend increase, indicating that chloroform solvent vapor provides the necessary energy for molecular motion, enabling molecular reconfiguration and recrystallization. Under a nonpolar solvent CS_2_ vapor with higher saturation vapor pressure and lower solubility, the PY‐IT absorption peak exhibited a red shift, whereas during the SVA process with a polar solvent CF vapor with lower saturation vapor pressure and higher solubility, the peak shows a blue shift. This suggests that polar solvents exert a stronger influence on the polymer acceptor, leading to a blue shift and the formation of a more compact hole transport layer, which enhances hole transport efficiency. The in situ GIWAXS findings suggest that the SVA‐induced morphological evolution can be divided into three stages: solvent swelling, recrystallization, and molecular rearrangement. An appropriate duration of solvent annealing promotes stable crystallinity, resulting in reduced d‐spacing, increased CCL, and optimized phase separation sizes, effectively enhancing PCE. Under the combined TA+CF treatment, the blade‐coated PM6: PY‐IT devices achieved a high PCE of 15.42%, comparable to that of spin‐coated devices without post‐treatment. However, excessive SVA may lead to crystal reswelling during molecular rearrangement, which is detrimental to performance improvement, whereas TA pretreatment helps to suppress this reswelling.

This study revealed a three‐stage mechanism (swelling‐recrystallization‐rearrangement) for SVA to dynamically regulate the morphology of all‐polymer blended films, and points out that polar solvents can optimize molecular stacking while non‐polar solvents promote phase separation, which provides a synergistic optimization strategy of process‐materials for the development of high‐performance scratch‐coated devices; and provides a basis for the subsequent study of multi‐solvent synergistic annealing, precision regulation of the dynamic process, and the development of a green process, which will promote the industrial application of large‐area flexible devices. It provides guidance for the subsequent research on multi‐solvent co‐annealing, precise regulation of dynamic processes and green process development, and promotes the industrialization of large‐area flexible devices.

## Conflict of Interest

The authors declare no conflict of interest.

## Supporting information



Supporting Information

## Data Availability

The data that support the findings of this study are available from the corresponding author upon reasonable request.
