# Supplementary material for: Real‐Time Probing of Morphological Evolution and Recrystallization During Solvent Annealing in Blade‐Coated All‐Polymer Organic Solar Cells Using In Situ X‐Ray Scattering
Source: Adv Sci (Weinh). 2025 Jun 23;12(35):e01823. doi: 10.1002/advs.202501823 (PMC12463108; doi:10.1002/advs.202501823)
Supplement: Supplementary file 1 — Supporting Information [file ADVS-12-e01823-s001.docx]

Supporting Information

Real-time probing of morphological evolution and recrystallization during solvent annealing in blade-coated all-polymer organic solar cells using in-situ X-ray scattering

Jialiang Hao ^1,2,†^, Yang Feng ^1,3, †^, Qianyi Ma ^1,4, †^, Hongxiang Li^5†^, Chunxia Hong ^1,6^, Chen Hou ^1^, Ying Wang ^1,6^,Yang Jing ^1,2^, Yiwen Li ^1^, Guangfeng Liu ^1^, Xiuhong Li ^1,6,7^, Aiguo Li ^1,6,7^, Fenggang Bian ^1,6,7^, Ruijie Ma *^8^, Yuanyang Wang*^2^, Yuying Huang ^1,6,7^, Chunming Yang*^1,6,7^

^1^Shanghai Synchrotron Radiation Facility, Shanghai Advanced Research Institute, Chinese Academy of Sciences, Shanghai, 201204, China

^2^School of Chemical Engineering and Technology, Taiyuan University of Science and Technology, Taiyuan, 030027, China

^3^Institute of Flexible Electronics, Northwestern Polytechnical University, Xi'an, 710100, China

^4^School of Materials Science and Engineering, University of Science and Technology Beijing, Beijing, 100049, China

^5^College of Polymer Science and Engineering State Key Laboratory of Polymer Materials Engineering, Sichuan University, Chengdu ,610065, China

^6^Shanghai Institute of Applied Physics, Chinese Academy of Sciences, Shanghai, 201800, China

^7^University of Chinese Academy of Sciences, Beijing, 100049, China

^8^Department of Electrical and Electronic Engineering, Research Institute for Smart Energy (RISE), Photonic Research Institute (PRI), The Hong Kang Polytechnic University, Hong Kang, 999077, China

Correspondence

Chunming Yang, Shanghai Synchrotron Radiation Facility, Shanghai Advanced Research Institute, Chinese Academy of Sciences, Shanghai, 201204 China.

Email: [yangcm@sari.ac.cn](mailto:yangcm@sari.ac.cn)

Yuanyang Wang, School of Chemical Engineering and Technology, Taiyuan University of Science and Technology, Taiyuan, 030027, China

Email: [wangyy@tyust.edu.cn](mailto:wangyy@tyust.edu.cn)

Ruijie Ma, Department of Electrical and Electronic Engineering, Research Institute for Smart Energy (RISE), Photonic Research Institute (PRI), The Hong Kang Polytechnic University, Hong Kang, 999077, China

Email: [rujie.ma@polyu.edu.hk](mailto:rujie.ma@polyu.edu.hk)

1. **Materials**

PM6, PY-IT, and PDINN were received from Dethon Optoelectronics Materials Science Technology Co., Ltd. Chloroform were received from Shanghai Hongbai Technology Co., LTD. Carbon disulfide were received from Tixiai (Shanghai) Chemical Industry Development Co., LTD. All of the materials were used as received without further purification. Indium Tin Oxide (ITO)-coated glass substrates were purchased from Advanced Election Technology Co., Ltd. Chlorobenzene (CB, anhydrous, 99.8%), Chloronaphthalene (CN, anhydrous, 99.8%), and methanol (anhydrous, 99.8%) were purchased from Sigma Aldrich. Chloroform (CF, anhydrous, 99.99%) was purchased from Kono science and Carbon disulfide (CS_2_, anhydrous, 98.0%) was purchased from TCI chemicals.

1. **Device Fabrication**

The APSC devices were fabricated with the conventional configuration of ITO/PEDOT: PSS/PM6:PY-IT/PDINN/Ag. The patterned ITO-coated glass substrate was cleaned with detergent and deionized water ultrasonic cleaning 30 min, then continuously cleaned three times with acetone, and isopropanol for 30 min of each step. After that the substrate was dried with a nitrogen gun and then placed in an ultraviolet-ozone cleaning machine for 30 min. This step can further remove the organic residue on the substrate surface and improve the hydrophilicity of the substrate surface. The PEDOT: PSS film was spin-coating on the treated ITO substrates with a thickness of about 40 nm and baked at 100 °C for 10 min. And then the treated substrates were then transferred into the glovebox for active layer deposition. The PM6:PY-IT solution was prepared by dissolving blends with a weight ratio of 1:1.2 in chlorobenzene (total concentration 15.4 mg/mL), and stirred at 50 ℃ for 24 hours. Chloronaphthalene was introduced into the solution 30 min prior to the application of the coating. The active layer solutions were coated on top of PEDOT layer using a doctor-blade coater (Suzhou Kejingte Intelligent Technology Co., Ltd, SPZ60) in air at a coating speed of 40 mm/s. The hotplate temperature was set as room temperature and the relative humidity was 40%. The shearing plate is silicon. The gap between the substrate and the shearing plate was 100 μm and the blade angle was 45°. The blade-coating started immediately after the solution dropped on the PEDOT: PSS layer, with an interval of about 2-3 s. The active layer was scratch coated on the PEDOT: PSS film at 40mm/s. Thermal annealing is to anneal the scraped and coated active layer on a hot table at 100 ° C for 10min in a glove box. Solvent annealing involves placing the scraped active layer device in a petri dish, dropping 200 ul of CF or CS_2_ solvent around it, and quickly closing the lid. Subsequently, a PDINN solution (in IPA, 1 mg/mL) was spin-coated at 3000 rpm for 30s to form the electron transfer layer. Finally, the 100 nm Ag was deposited as anode below the vacuum level of 4.0 × 10^-4^ Pa.

1. **The saturated vapor pressures and solubility measurement**

The Antoine coefficients of CF and CS_2_ solvents by consulting relevant data (Hydrocarbon Processing, Vol.68 No.10 P65-68, 1989), as shown in Table S1. The saturated vapor pressure of the solvent can be estimated using the Antoine formula: $\log_{10}p=A-B(T+C)$

where A, B, and C are constants of vapor pressure, P is the vapor pressure of the substance (mmHg), T is temperature (℃).

Based on the temperature range of the material vapor pressure constant, we choose to calculate the saturated vapor pressures of CS_2_ and CF using the Antoine formula at 50 ℃. The results shown in Table S1 show that the saturated vapor pressures of the four solvents meet the following order: CS_2_ > CF, but the boiling point order is: CS_2_ < CF.

The solubilities of PM6 and PY-IT in CF and CS_2_ were measured by an optical method. ^[1]^First, a series of standard absorption spectra of pm6 and PY-IT were recorded in CF and CS_2_. The optical density showed a linear relationship on concentration and the absorption coefficient could be obtained. Next, the saturated solutions were prepared by adding the excessive materials followed by heating and cooling down to room temperature. Then, the saturated solution was filtered through a PTFE syringe filter and diluted with 100 to 10000 volumes. Finally, measuring the absorption of diluted solution, the solubility of materials can be obtained after calculation, as shown in Table S2 and Figure S1.


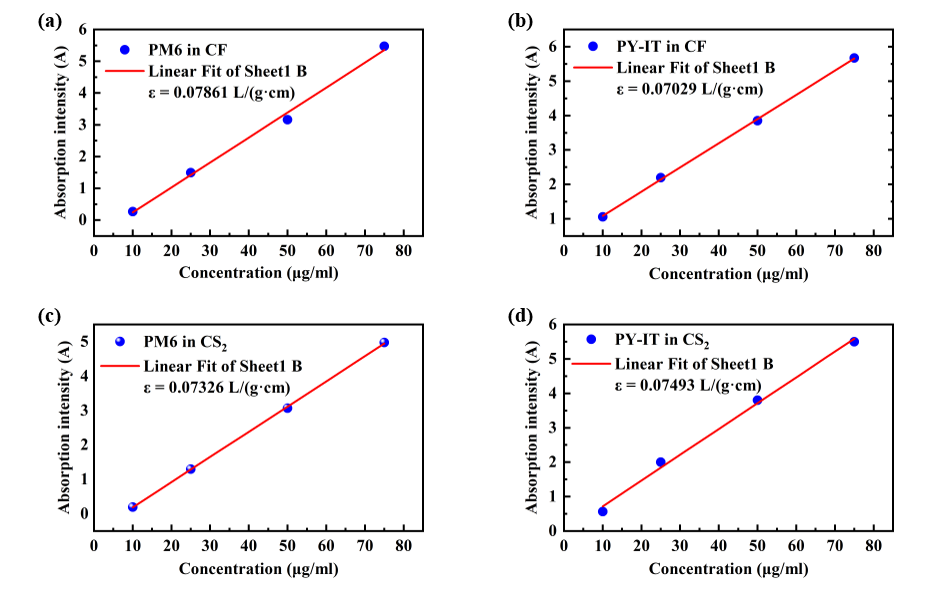


**Figure S1** Relationship of absorption intensity on solution concentration.

**Table S1** Antoine coefficients and applicable temperature range for CF and CS_2_.

| **Solvent** | **A** | **B** | **C** | **Lowest temperature** | **Maximum temperature** |
| --- | --- | --- | --- | --- | --- |
| **CF** | 6.9 | 1163.0 | 227.4 | -30 | 150 |
| **CS_2_** | 6.9 | 1122.5 | 236.5 | -10 | 160 |

**Table S2** Saturated vapor pressure and boiling point statistics for CS_2_ and CF.

| **Solvent** | **Vapor pressure (mmHg)/50℃** | **Boiling point/℃** | **Solubility/mg/mL** | |
| --- | --- | --- | --- | --- |
|  |  |  | **PM6** | **PY-IT** |
| **CF** | 513.7 | 61.2 | 27.0 | 102.6 |
| **CS_2_** | 856.9 | 46.2 | 22.6 | 79.5 |

1. **The calculation method of *δ*_d_, *δ*_p_ and *δ*_h_**

In the main text, these equations 1 and 2 contain four additive molar functions, some auxiliary formulas, and final expressions for each component of *δ_t(total)_* and *δ*. The incremental values of the molar attraction functions are given in Table S3 and S4. *F_t_* is the molar attraction function, *F_p_* is the polar component, *V* is the molar volume of the polymer structural unit, and *∆_T_ ^(P)^* is the correction value used in the auxiliary equations.^[2,3]^

The calculation process was as follows:

**Additive molar functions:**

$F_{t}=\Sigma N_{i}F_{t,i}$ (S1)

$F_{P}=\Sigma N_{i}F_{p,i}$ (S2)

$V=\Sigma N_{i}V_{i}$ (S3)

**Auxiliary equations**:

$\alpha(P)=777\Delta_{T}^{(P)}/V$ (S4)

$n=0.5/\Delta_{T}^{(P)}$ (S5)

**Expressions for *δ* and *δ* components**:

$\delta_{t}=\left(F_{t}+B/n\right)/V$ (S6)

$B=basevalue=277$ (S7)

$\delta_{p}=\delta_{t}\left(\frac{1}{\alpha^{(P)}}\frac{F_{P}}{F_{t}+B/n}\right)^{1/2}$ (S8)

$\delta_{h}=\delta_{t}\left[\left(\alpha^{(P)}-1\right)/\alpha^{(P)}\right]^{1/2}$ (S9)

$\delta_{d}=\left(\delta_{t}^{2}-\delta_{p}^{2}-\delta_{h}^{2}\right)^{1/2}$ (S10)

**Table S3.** *F_t_*, *F_p_*, *V*, and *∆_T_ ^(P)^* of the structural units in PM6 and PY-IT**.**

| Groups | *F_t,I_* ((MJ/m^3^)^1/2^/mol) | *F_p,i_* ((MJ/m^3^)^1/2^/mol) | *V_i_* (cm^3^/mol) | *Δ_T,i_^(P)^* |
| --- | --- | --- | --- | --- |
| -CH_3_ | 303.5 | 0.0 | 21.55 | 0.022 |
| -CH_2_- | 269.0 | 0.0 | 15.55 | 0.020 |
| >CH- | 176.0 | 0.0 | 9.56 | 0.012 |
| >C< | 65.5 | 0.0 | 3.56 | 0.040 |
| CH_ar_ | 241.0 | 62.5 | 13.42 | 0.018 |
| C_ar_ | 201.0 | 65.0 | 7.42 | 0.015 |
| -CH= | 249 | 59.5 | 13.18 | 0.0185 |
| >C= | 173 | 63 | 7.18 | 0.013 |
| -F | 845 | 73.5 | 11.2 | 0.006 |
| >C=O | 538 | 525 | 17.3 | 0.04 |
| -C≡N | 725 | 725 | 23.1 | 0.06 |
| >N- | 125.0 | 125.0 | 12.60 | 0.009 |
| -S- | 428.0 | 428.0 | 18.00 | 0.032 |

**Table S4.** Solubility parameters (*δ_d_*, *δ_p_*, and *δ_h_*) of PM6、PY-IT、CF and CS_2_.

| Solvents | CF | CS_2_ | PM6 | PY-IT |
| --- | --- | --- | --- | --- |
| *δ_d_* (mPa^1/2^) | 17.8 | 20.5 | 17.3 | 15.3 |
| *δ_p_* (mPa^1/2^) | 3.1 | 0 | 11.3 | 11.3 |
| *δ_h_* (mPa^1/2^) | 5.7 | 0.6 | 7.9 | 7.1 |
| *R_a_*  CF | \ | \ | 8.6 | 9.7 |
| *R*_a_ CS_2_ | \ | \ | 14.9 | 16.7 |
| △*δ_d_*  CF | \ | \ | 0.5 | 2.5 |
| △*δ_d_*  CS_2_ | \ | \ | 3.2 | 5.2 |
| △*δ_P_* CF | \ | \ | 8.2 | 8.2 |
| △*δ_P_* CS_2_ | \ | \ | 11.3 | 11.3 |
| △*δ_h_*  CF | \ | \ | 2.2 | 1.4 |
| △*δ_h_* CS_2_ | \ | \ | 7.3 | 6.5 |

1. **In situ UV-vis absorption measurement**

For the UV-vis absorption spectra experiment, the light source and detector of the Ocean Insight DH-2000-BAL spectrometer are fixed above and below and the light was introduced into the customized chamber through a hole in was adhered to the sample holder by mechanical pump. The spectra were recorded between 200 and 1000 nm, with a wavelength step of 0.05 nm, an exposure time of 0.5 s per frames. The UV-vis absorption spectra are calculated from the transmission spectra according to the equation $A_{\lambda}=-\log_{10}(T)$, where 𝐴_λ_ is the absorbance at a certain wavelength (λ) and T is the calculated transmittance. Samples were prepared on Glass substrates using identical blend solutions and processing conditions as those used in devices.


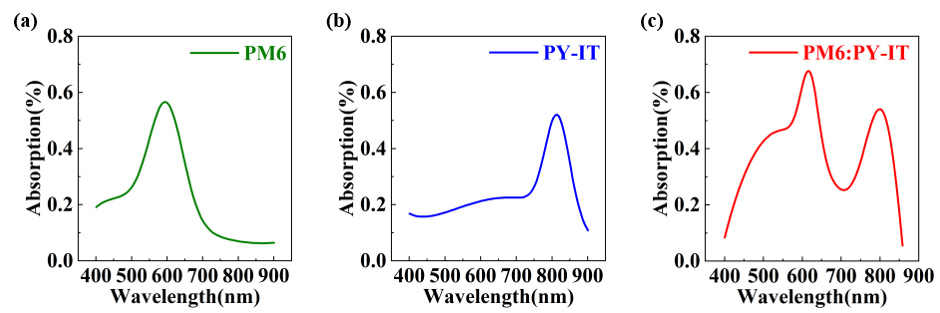


**Figure S2.** Absorption peak location of pure PM6, pure PY-IT and PM6:PY-IT blend films.
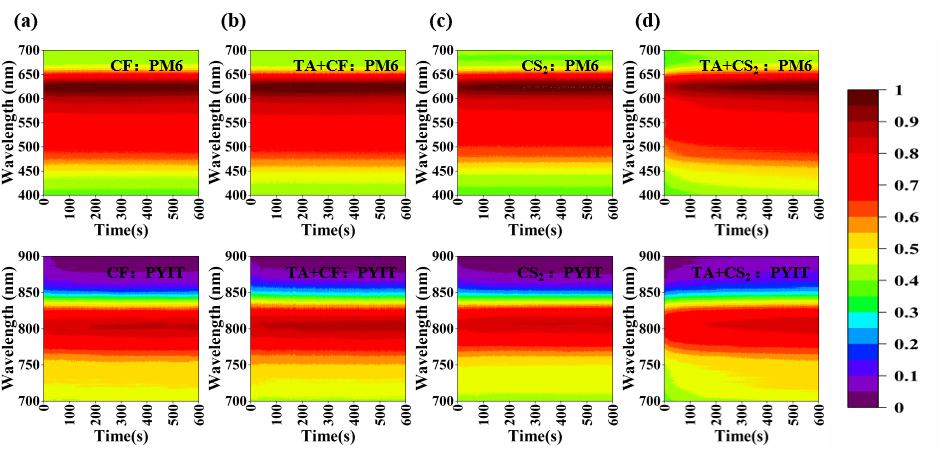


**Figure S3.** Contour map of PM6:PY-IT films treated with CF, TA+CF, CS_2_, and TA+CS_2_.

1. **Morphology Characterizations**

**Atomic Force Microscopy (AFM) Imaging**

Topographic images of the films were obtained from a Guangzhou original CSPM5500 atomic force microscopy (AFM) with the type of dimension edge with Scan Asyst in the tapping mode using an etched silicon cantilever at a nominal load of ~2 nN, the scanning rate for a 2 μm×2 μm image size was 1.0 Hz.

**Transmission Electron Microscopy (TEM) Characterization**

Films were spin-coated on PEDOT: PSS-coated silicon substrates. The films were floated off the substrates in deionized water and collected on lacey carbon coated TEM grids (Electron Microscopy Sciences). TEM studies were performed a JEOL JEM-F200 TEM equipped with an electron monochromator and a Gatan Imaging Filter (GIF) Quantum 966.

**Table S5.** Surface roughness of blend films from AFM measurements.

| **Treatment** | **As Cast** | **TA** | **CF** | **TA+CF** | **CS_2_** | **TA+CS_2_** |
| --- | --- | --- | --- | --- | --- | --- |
| *R_q_* (nm) | 3.07 ± 0.05 | 3.05 ± 0.08 | 2.77 ± 0.06 | 2.56 ± 0.05 | 2.94 ± 0.04 | 2.72 ± 0.07 |

1. **GIWAXS and GISAXS Measurements**

GIWAXS and GISAXS was carried out on beamline BL16B1 at the Shanghai Synchrotron Radiation Facility (SSRF). The beam line has two detectors (PILATUS 900K for GIWAXS and PILATUS 2M detector for GISAXS, with a pixel size of 0.712 mm × 0.172 mm) installed in-line downstream of the sample to collect successively X-ray scattering data simultaneously. The distance from the sample to the detector set to 255 mm and 2114 mm. An incident photon energy of 10 keV was applied, with a corresponding wavelength of 0.124 nm. An incident angle αi of 0.15° (near the critical angle) was applied in the GIWAXS/GISAXS experiment, thus providing a global and strong (averaged) scattering signal for the sample. ^[4,5]^ The Offline GIWAXS/GISAXS data are collected with an exposure time of 30 s. Samples were prepared on Si substrates using identical blend solutions and processing conditions as those used in devices. Si wafers make ideal substrates because they are very smooth at the atomic/nano scale, and are also very flat across larger (macroscale) distances, which is beneficial for GIWAXS characterization.

**In-situ GIWAXS Characterization.** In-situ GIWAXS measurements were performed at beamline BL16B1 of SSRF. The distance from the sample to the detector (SDD) was 255 mm, and the X-ray energy was 10 keV (wavelength λ=1.24 Å). Synchrotron X-ray continuously exposes on substrate with an incident angle of 0.15° and exposure time of 4.9 s. The detector pixel size is 0.172 mm × 0.172 mm. Samples were prepared on Si substrates using identical blend solutions and processing conditions as those used in devices. One dimensional experimental data were obtained with the SGTools software package programmed by Zhao et al.^[6]^

**Data analysis.** The structural information of blend films such as the period of arrangement and lamellar stacking spacing is obtained via the Bragg equation, as well as the crystal coherence length (CCL) can be obtained from the Scherrer formula,^[7]^ and the specific expressions of the Bragg equation and Scherrer formula are as follows.

$d=\frac{\lambda}{2sin(\theta)}=\frac{2\pi}{q}$ (S13)

$CCL=\frac{K\lambda}{FWHM\cdot cos(\theta)}$ (S14)

where d is the lamellar stacking spacing, and CCL is the crystal domain along the specified direction called crystal coherence length, which is generally considered to be equivalent to the grain size. 𝜆 is the value of X-ray wavelength; K is a dimensionless shape factor,^[8]^ generally taken as K = 0.89, FWHM refers to the full width at half maximum of the scattering peak, θ is the scattering angle.

**GISAXS fitting model.** In our calculation, for the blend films of PM6:PY-IT, a universal model based on the Distorted Wave Born Approximation (DWBA) was used with fitting equations are as follows^[9,10]^:

$I(q)=\frac{A_{1}}{{[1+(q\xi)^{2}]}^{2}}+A_{2}\langle P(q,R)\rangle S(q,R,\eta,D)+B$ (S14)

where *q* is the scattering vector; A1, A2, and B are independent fit parameters proportional to the overall strength of each term. The first term is the Debye Andersonese Brumberger (DAB) term, which describes the scattering from intermixing domains, and *ξ* is the average coherence length of the donor phase in this study. The second term is the scattering intensity contribution from the pure domains of the molecules in the mixtures, where 𝑃(𝑞, 𝑅) is the shape factor of the pure domains, and a spherical model with an average radius of R is used in the present experiment with the following expression:

$\begin{matrix} F_{sp}(q,R) & =4\pi R^{3}\frac{sin(qR)-qRcos(qR)}{(qR)^{3}}e^{iq_{z}R} \\ & V_{sp}=\frac{4}{3}\pi R^{3},S_{sp}=\pi R^{2},R_{sp}=R. \end{matrix}$ (S15)

𝑆 (𝑞, 𝑅, 𝜂, 𝐷) is the structure factor of pure domains, which is generally modeled using the fractal network model to characterize the degree of aggregation and morphology of small molecules in the film of the blends with the following expression^[11]^:

$S(q)=1+\frac{sin[(D-1)tan^{-1}(q\eta)]}{(qR)^{D}}\cdot\frac{D\Gamma(D-1)}{[1+\frac{1}{(q\eta)^{2}}]^{\frac{(D-1)}{2}}}$ (S16)

where *R* is the average radius in the shape factor; *η* denotes the coherence length of the fractal network, and *D* is the fractal dimension. The average pure domain size of a small molecule can be estimated by the fractal network radius of gyration, *R_g_*, with the following equation:

$R_{g}=[\frac{D\left( D+1 \right)}{2}]^{\frac{1}{2}} \eta$ (S17)


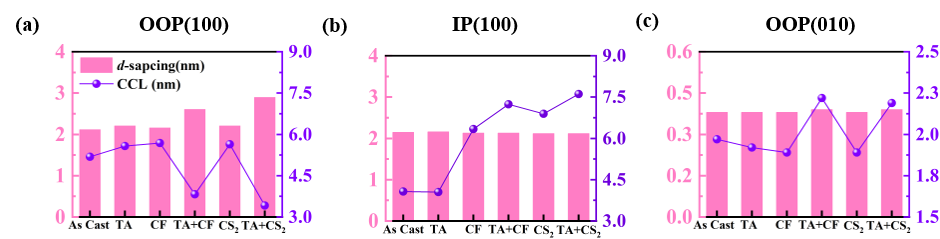


**Figure S4.** Calculated *d*-spacing and CCL values for (a) OOP(100), (b) IP(100) and (c) OOP(010) planes.


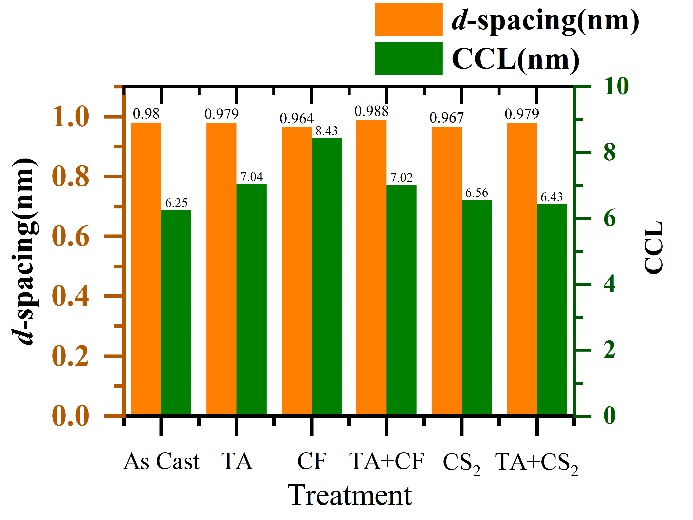


**Figure S5.** The *d*-spacing and CCL of the second-order reflection peak near *q* = 6.2 nm^-1^ in the in-plane direction

**Table S6.** Fitting data obtained from GIWAXS under different treatment conditions.

| **Treatment** | **Peak** | **Peak position**  **(nm^-1^)** | ***d*-spacing**  **(nm)** | **FWHM**  **(nm^-1^)** | **CCL**  **(nm)** |
| --- | --- | --- | --- | --- | --- |
|  | (100) OOP | 2.97  (2.97±0.01) | 2.12  (2.12±0.01) | 1.09  (1.09±0.02) | 5.19  (5.19±0.12) |
| **As Cast** | (100) IP | 2.93  (2.93±0.06) | 2.15  (2.15±0.04) | 1.39  (1.39±0.16) | 4.07  (4.07±0.43) |
|  | (010) OOP | 16.38  (16.38±0.03) | 0.38  (0.38±0.01) | 2.87  (2.87±0.11) | 1.97  (1.97±0.07) |
|  | (100) OOP | 2.84  (2.84±0.01) | 2.21  (2.21±0.01) | 1.10  (1.10±0.02) | 5.58  (5.58±0.13) |
| **TA** | (100) IP | 2.92  (2.92±0.06) | 2.16  (2.16±0.04) | 1.40  (1.40±0.16) | 4.05  (4.05±0.42) |
| **CF** | (010) OOP | 16.42  (16.42±0.03) | 0.38  (0.38±0.01) | 2.95  (2.95±0.13) | 1.92  (1.92±0.08) |
|  | (100) OOP | 2.91  (2.91±0.04) | 2.16  (2.16±0.03) | 0.99  (0.99±0.01) | 5.69  (5.69±0.06) |
|  | (100) IP | 2.95  (2.95±0.07) | 2.13  (2.13±0.02) | 0.89  (0.89±0.05) | 6.34  (6.34±0.03) |
|  | (010) OOP | 16.39  (16.39±0.03) | 0.38  (0.38±0.01) | 3.00  (3.00±0.13) | 1.89  (1.89±0.07) |
| **TA + CF** | (100) OOP | 2.78  (2.78±0.10) | 2.26  (2.26±0.16) | 1.13  (1.13±0.34) | 4.99  (4.99±0.73) |
|  | (100) IP | 2.92  (2.92±0.03) | 2.15  (2.15±0.02) | 1.32  (1.32±0.09) | 4.26  (4.26±0.18) |
|  | (010) OOP | 16.42  (16.42±0.04) | 0.38  (0.38±0.01) | 3.00  (3.00±0.18) | 1.89  (1.89±0.47) |
|  | (100) OOP | 2.85  (2.85±0.01) | 2.21  (2.21±0.08) | 1.00  (1.00±0.03) | 5.64  (5.64±0.17) |
| **CS_2_** | (100) IP | 2.94  (2.94±0.01) | 2.13  (2.13±0.03) | 0.82  (0.82±0.05) | 6.89  (6.89±0.48) |
|  | (010) OOP | 16.44  (16.44±0.02) | 0.38  (0.38±0.01) | 2.99  (2.99±0.14) | 1.89  (1.89±0.04) |
|  | (100) OOP | 2.82  (2.82±0.15) | 2.23  (2.23±0.13) | 1.00  (1.00±0.02) | 5.65  (5.65±0.07) |
| **TA + CS_2_** | (100) IP | 2.96  (2.96±0.05) | 2.12  (2.12±0.09) | 0.93  (0.93±0.12) | 6.09  (6.09±0.03) |
|  | (010) OOP | 16.44  (16.44±0.02) | 0.38  (0.38±0.01) | 2.90  (2.90±0.08) | 1.95  (1.95±0.07) |


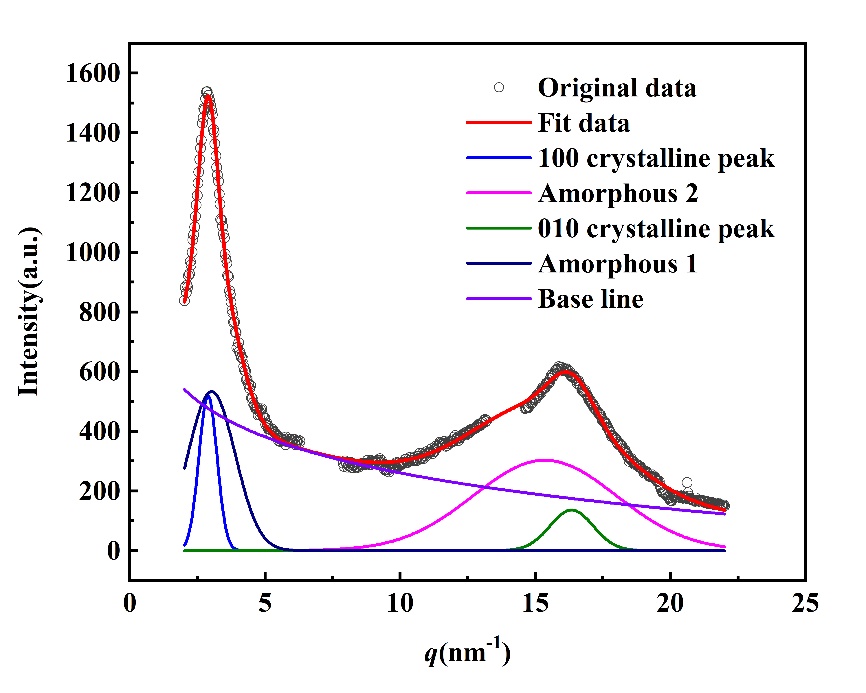


**Figure S6.** Typical schematic of crystallinity and amorphous index from peak fitting

**Table S7**. Crystallinity and the ratio of amorphous phase obtained from GIWAXS

| **Treatment** | **As Cast** | **TA** | **CF** | **TA+CF** | **CS_2_** | **TA+CS_2_** |
| --- | --- | --- | --- | --- | --- | --- |
| Crystalline  Area | 867.9  (867.9 ± 5.8) | 935.2  (935.2 ± 8.6) | 1144.1  (1144.1 ± 7.6) | 1259.8  (1259.8 ± 19.2) | 1080.4  (1080.4 ± 15.9) | 1184.1  (1184.1 ± 14.4) |
| Amorphous  Area | 3174.1  (3174.1 ± 23.2) | 3071.6  (3071.6 ± 11.8) | 2891.9  (2891.9 ± 44.5) | 2801.0  (2801.0 ± 8.9) | 2983.3  (2983.3 ± 24.3) | 2833.1  (2833.1 ± 28.6) |
| Crystallinity | 21.5  (21.5 ± 0.1) | 23.3  (23.3 ± 0.2) | 28.3  (28.3 ± 0.2) | 31.0  (31.0 ± 0.5) | 26.6  (26.6 ± 0.4) | 29.5  (29.5 ± 0.4) |
| Amorphous  Ratio (%) | 78.5  (78.5 ± 1.9) | 76.7  (76.7 ± 1.9) | 71.7  (71.7 ± 1.9) | 69.0  (69.0 ± 1.7) | 73.4  (73.4 ± 1.8) | 70.5  (70.5 ± 1.8) |


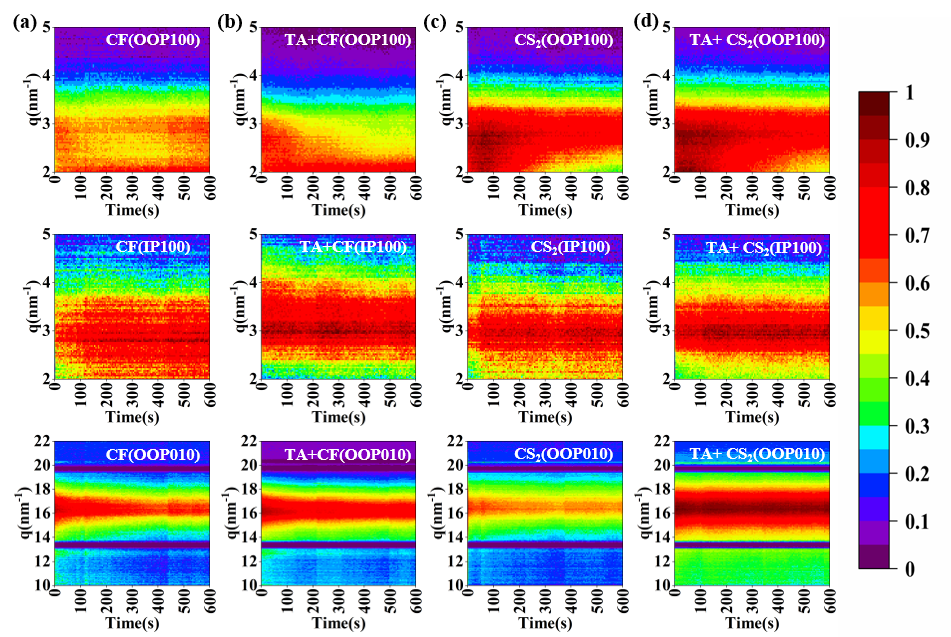


**Figure S7.** Contour maps of OOP(100), IP(100) and OOP(010) peaks of PM6:PY-IT films treated by CF, TA+CF, CS_2_ and TA+CS_2_.


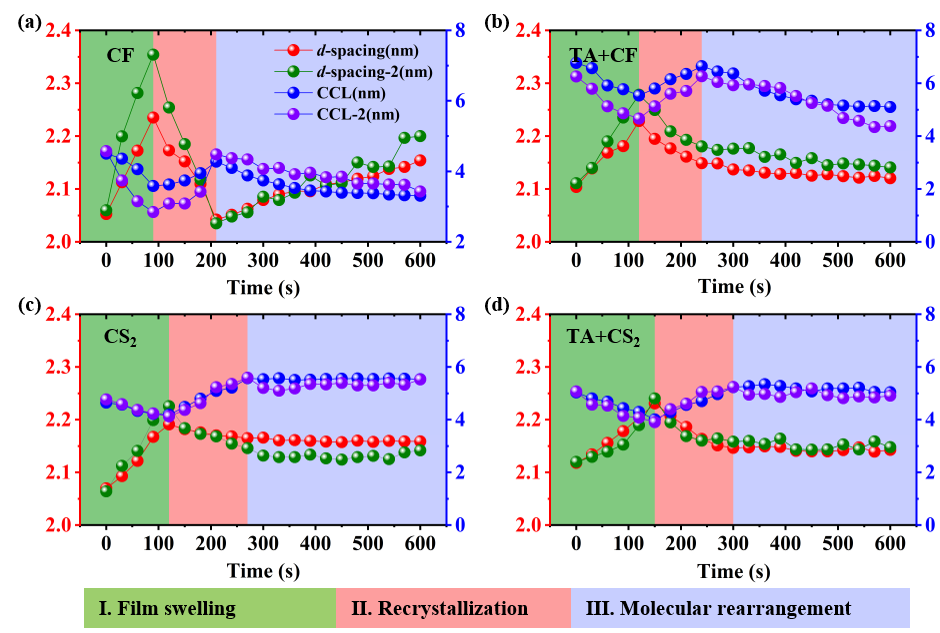


**Figure S8.** Evolution of *d*-spacing and CCL evolution of (OOP)100 peak for blade coated PM6: PY-IT blend films with a) CF, b) TA+ CF, c) CS_2_, and d) TA+ CS_2_. Data were obtained from in-situ GIWAXS measurements.


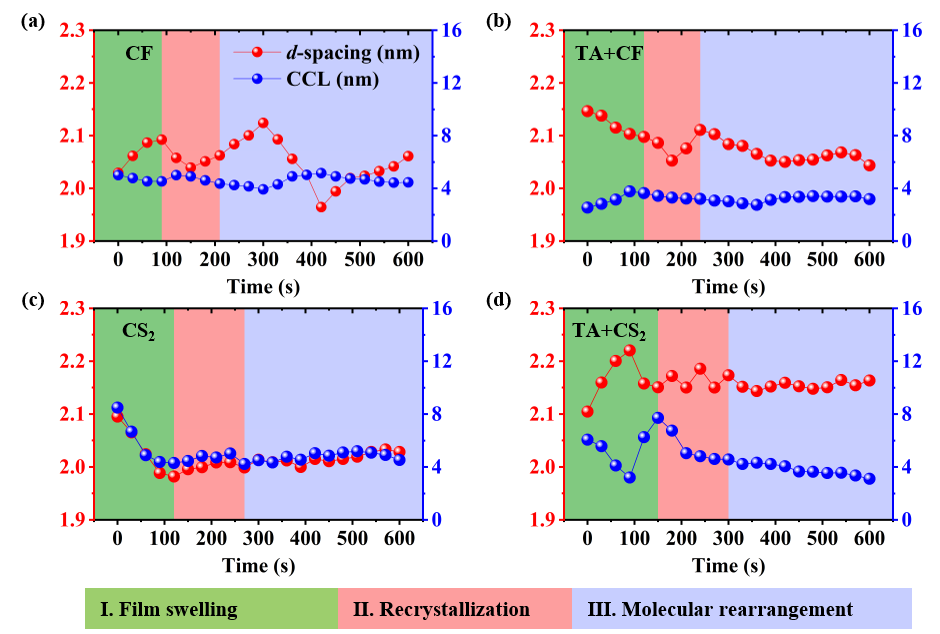


**Figure S9.** The time-dependent d-spacing and CCL evolution of (IP) 100 peak for blade coated PM6: PY-IT blend films with a) CF, b) TA+ CF, c) CS_2_, and d) TA+ CS_2_. The data were obtained from in situ GIWAXS measurements.


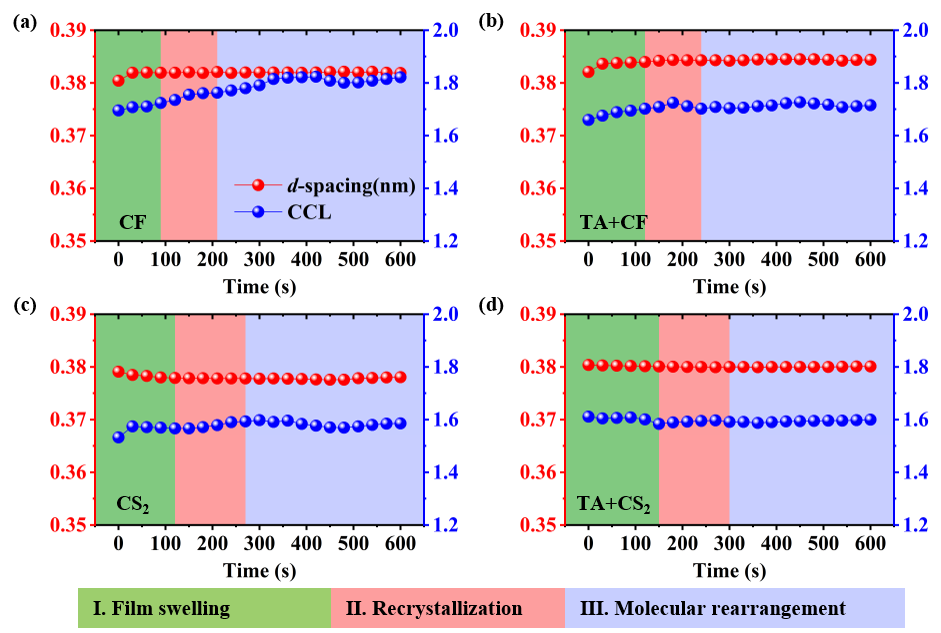


**Figure S10.** The time-dependent d-spacing and CCL evolution of OOP (010) peak for blade coated PM6: PY-IT blend films with a) CF, b) TA+ CF, c) CS_2_, and d) TA+ CS_2_. The data were obtained from in situ GIWAXS measurements.


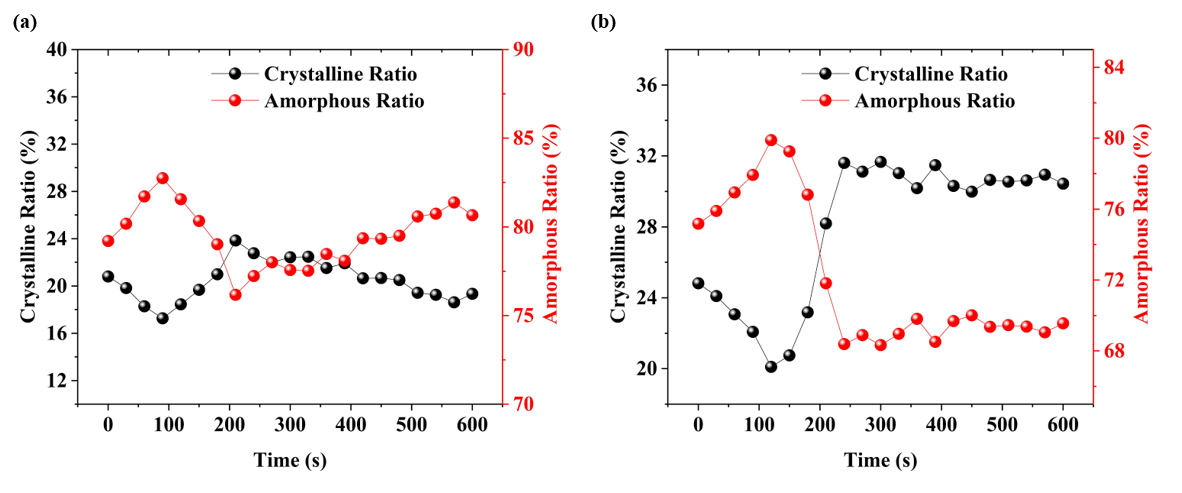


**Figure S11.** Trends of crystalline and amorphous phases calculated by in situ GIWAXS under (a) CF and (b) TA+CF treatment

**Table S8.** The relative path of crystal orientation is obtained from the ratio of two peaks of (010) (χ = 90°~ 135° and 135°~ 180°).

| **Treatment** | **Face-on area** | **Edge-on area** | **Face-on/All area** | **Edge-on/All area** |
| --- | --- | --- | --- | --- |
| **As Cast** | 29494 | 14053 | 0.677 | 0.323 |
| **TA** | 33617 | 17306 | 0.660 | 0.340 |
| **CF** | 35310 | 17415 | 0.670 | 0.330 |
| **TA+CF** | 32869 | 17766 | 0.649 | 0.351 |
| **CS_2_** | 27286 | 12223 | 0.691 | 0.309 |
| **TA+CS_2_** | 26822 | 10844 | 0.712 | 0.288 |

**Table S9**. Fitting parameters derived from GISAXS analysis.

| **Treatment** | **Domain size ξ**  **[nm]** | **Fractal dimension D** | **Guinier radius 2Rg [nm]** | **Acceptor correlation length η[nm]** |
| --- | --- | --- | --- | --- |
| **As Cast** | 26.5  (26.5±0.3) | 2.9 | 28.44  (28.44±0.09) | 3.98  (3.98±0.04) |
| **TA** | 27.4  (27.4±0.4) | 2.9 | 28.93  (28.93±0.03) | 6.02  (6.02±0.06) |
| **CF** | 30.6  (30.6±0.2) | 2.9 | 29.77  (29.77±0.06) | 6.26  (6.26±0.05) |
| **TA+CF** | 32.8  (32.8±0.3) | 2.9 | 32.91  (32.91±0.03) | 6.96  (6.96±0.07) |
| **CS_2_** | 30.4  (30.4±0.2) | 2.9 | 29.34  (29.34±0.05) | 6.17  (6.17±0.03) |
| **TA+CS_2_** | 31.2  (31.2±0.1) | 2.9 | 31.01  (31.01±0.02) | 6.52  (6.52±0.08) |

1. **PCE and EQE measurements**

The J-V characteristics were performed in N2-filled glovebox under AM 1.5G (100 mW/cm^2^) by using a Keysight B2901A SMU source meter unit and an AAA solar simulator (SS-F7-3A, Enli Technology CO., Ltd.) calibrated by a standard Si photovoltaic cell. The external quantum efficiency (EQE) was measured by a certified incident photon to electron conversion (IPCE) equipment (QE-R) from JINZHU Technology Co., Lt. The light intensity at each wavelength was calibrated using a standard monocrystalline Si photovoltaic cell.

**Table S10.** The photovoltaic data of the PM6:PY-IT devices with CF solvents and treating times. All data were obtained under AM 1.5G (100mW cm^-2^) illumination in conventional devices.

| **Treatment** | **Annealing time(s)** | **Voc**  **(V)** | **Jsc**  **(mA cm^-2^)** | **FF**  **(%)** | **^a)^PCE(Average)**  **(%)** |
| --- | --- | --- | --- | --- | --- |
| **CF** | 30 | 0.92  (0.92±0.005) | 22.47  (22.47±0.36) | 65.21  (65.21±0.89) | 13.48  (13.48±0.05) |
|  | 60 | 0.92  (0.92±0.004) | 23.34  (23.34±0.45) | 66.16  (66.16±0.77) | 14.18  (14.18±0.17) |
|  | 90 | 0.93  (0.93±0.006) | 22.24  (22.24±0.58) | 70.72  (70.72±0.18) | 14.52  (14.52±0.12) |
|  | 120 | 0.93  (0.93±0.002) | 22.71  (22.71±0.15) | 68.61  (68.61±0.45) | 14.43  (14.43±0.41) |
|  | 150 | 0.93  (0.93±0.005) | 22.27  (22.27±0.56) | 68.64  (0.68±0.75) | 14.19  (14.19±0.22) |
|  | 180 | 0.93  (0.93±0.001) | 22.30  (22.30±0.09) | 67.27  (67.27±0.11) | 13.93  (13.93±0.15) |

**Table S11.** The photovoltaic data of PM6:PY-IT devices with TA+CF solvents and treating times. All data were obtained under AM 1.5G (100mW cm^-2^) illumination in the conventional devices.

| **Treatment** | **Annealing time(s)** | **Voc**  **(V)** | **Jsc**  **(mA cm^-2^)** | **FF**  **(%)** | **^a)^PCE(Average)**  **(%)** |
| --- | --- | --- | --- | --- | --- |
| **TA+CF** | 30 | 0.94  (0.94±0.01) | 21.62  (21.62±0.04) | 67.98  (67.98±0.18) | 13.81  (13.81±0.16) |
|  | 60 | 0.94  (0.94±0.01) | 22.25  (22.25±0.05) | 67.00  (67.00±0.08) | 13.98  (13.98±0.20) |
|  | 90 | 0.94  (0.94±0.01) | 23.53  (23.53±0.27) | 70.03  (70.03±0.39) | 15.42  (15.42±0.31) |
|  | 120 | 0.94  (0.94±0.01) | 21.63  (21.63±0.16) | 69.01  (69.01±0.09) | 13.95  (13.95±0.25) |
|  | 150 | 0.94  (0.94±0.01) | 21.13  (21.13±0.28) | 69.12  (69.12±0.11) | 13.54  (13.54±0.24) |
|  | 180 | 0.94  (0.94±0.01) | 20.60  (20.60±0.07) | 69.92  (69.92±0.22) | 13.40  (13.40±0.55) |

**Table S12.** The photovoltaic data of PM6:PY-IT devices with CS_2_ solvents and treating times. All data were obtained under AM 1.5G (100mW cm^-2^) illumination in the conventional devices.

| **Treatment** | **Annealing time(s)** | **Voc**  **(V)** | **Jsc**  **(mA cm^-2^)** | **FF**  **(%)** | **^a)^PCE(Average)**  **(%)** |
| --- | --- | --- | --- | --- | --- |
| **CS_2_** | 30 | 0.94  (0.94±0.01) | 22.17  (22.17±0.16) | 65.97  (65.97±0.41) | 13.70  (13.70±0.43) |
|  | 60 | 0.93  (0.93±0.01) | 22.01  (22.01±0.44) | 67.58  (67.58±0.06) | 13.85  (13.85±0.20) |
|  | 90 | 0.93  (0.93±0.01) | 22.47  (22.47±0.24) | 67.80  (67.80±0.16) | 14.35  (14.35±0.25) |
|  | 120 | 0.94  (0.94±0.01) | 21.78  (21.78±0.04) | 68.71  (68.71±0.61) | 13.94  (13.94±0.11) |
|  | 150 | 0.94  (0.94±0.01) | 21.30  (21.30±0.19) | 68.58  (68.58±0.13) | 13.79  (13.79±0.06) |
|  | 180 | 0.94  (0.94±0.01) | 20.41  (20.41±0.16) | 68.87  (68.87±0.35) | 13.29  (13.29±0.01) |

**Table S13.** The photovoltaic data of PM6:PY-IT devices with TA+CS_2_ solvents and treating times. All data were obtained under AM 1.5G (100mW cm^-2^) illumination in the conventional devices.

| **Treatment** | **Annealing time(s)** | **Voc**  **(V)** | **Jsc**  **(mA cm^-2^)** | **FF**  **(%)** | **^a)^PCE(Average)**  **(%)** |
| --- | --- | --- | --- | --- | --- |
| **TA+CS_2_** | 30 | 0.94  (0.94±0.01) | 20.97  (20.97±0.15) | 68.62  (68.62±0.48) | 13.57  (13.57±0.05) |
|  | 60 | 0.94  (0.94±0.01) | 21.20  (21.20±0.39) | 68.00  (68.00±0.16) | 13.60  (13.60±0.07) |
|  | 90 | 0.93  (0.93±0.01) | 23.29  (23.29±0.15) | 68.52  (68.52±0.25) | 14.78  (14.78±0.05) |
|  | 120 | 0.94  (0.92±0.01) | 22.14  (22.14±0.49) | 67.96  (67.96±0.12) | 14.06  (14.06±0.06) |
|  | 150 | 0.94  (0.94±0.01) | 21.70  (21.70±0.11) | 68.29  (68.29±0.20) | 13.98  (13.98±0.04) |
|  | 180 | 0.94  (0.94±0.01) | 21.89  (21.89±0.16) | 66.79  (66.79±0.10) | 13.78  (13.78±0.02) |

1. The average parameters were calculated over 10 independent cells.
2. **Reference**

[1] B. Walker, A. Tamayo, D. T. Duong, X.-D. Dang, C. Kim, J. Granstrom, T.-Q. Nguyen, *Advanced Energy Materials* **2011**, *1*, 221.

[2] L. Chen, K. Zhao, X. Cao, J. Liu, X. Yu, Y. Han, *Polymer* **2018**, *149*, 23.

[3] D. L. Ho, C. J. Glinka, *Journal of Polymer Science Part B: Polymer Physics* **2004**, *42*, 4337.

[4] X. Sun, K. Liu, N. Zhao, F. Bian, C. Yang, Y. Huang, *J. Phys. Chem. B* **2022**, *126*, 1625.

[5] X. Sun, W. Huang, C. Yang, F. Qi, J. Chen, N. Zhao, X. OuYang, *ACS Appl. Energy Mater.* **2023**, *6*, 6826.

[6] N. Zhao, C. Yang, F. Bian, D. Guo, X. Ouyang, *J Appl Crystallogr* **2022**, *55*, 195.

[7] A. Mahmood, J.-L. Wang, *Solar RRL* **2020**, *4*, 2000337.

[8] D.-M. Smilgies, *J Appl Crystallogr* **2009**, *42*, 1030.

[9] Y. Liu, J. Zhao, Z. Li, C. Mu, W. Ma, H. Hu, K. Jiang, H. Lin, H. Ade, H. Yan, *Nat Commun* **2014**, *5*, 5293.

[10] S. Yao, C. Huang, Q. Wang, T. Yang, S. Shi, Y. Liu, C. Zhao, Z. Zhang, X. Shen, T. Li, B. He, W. Lin, T. Zhang, B. Zou, T. Liu, *Solar RRL* **2022**, *6*, 2200617.

[11] H.-C. Liao, C.-S. Tsao, Y.-T. Shao, S.-Y. Chang, Y.-C. Huang, C.-M. Chuang, T.-H. Lin, C.-Y. Chen, C.-J. Su, U.-S. Jeng, Y.-F. Chen, W.-F. Su, *Energy Environ. Sci.* **2013**, *6*, 1938.
